# Supplementary figures and images for: Cost-effectiveness of hypertension therapy based on 2020 International Society of Hypertension guidelines in Ethiopia from a societal perspective
Source: PLoS One. 2022 Aug 29;17(8):e0273439. doi: 10.1371/journal.pone.0273439 (PMC9423649; doi:10.1371/journal.pone.0273439)

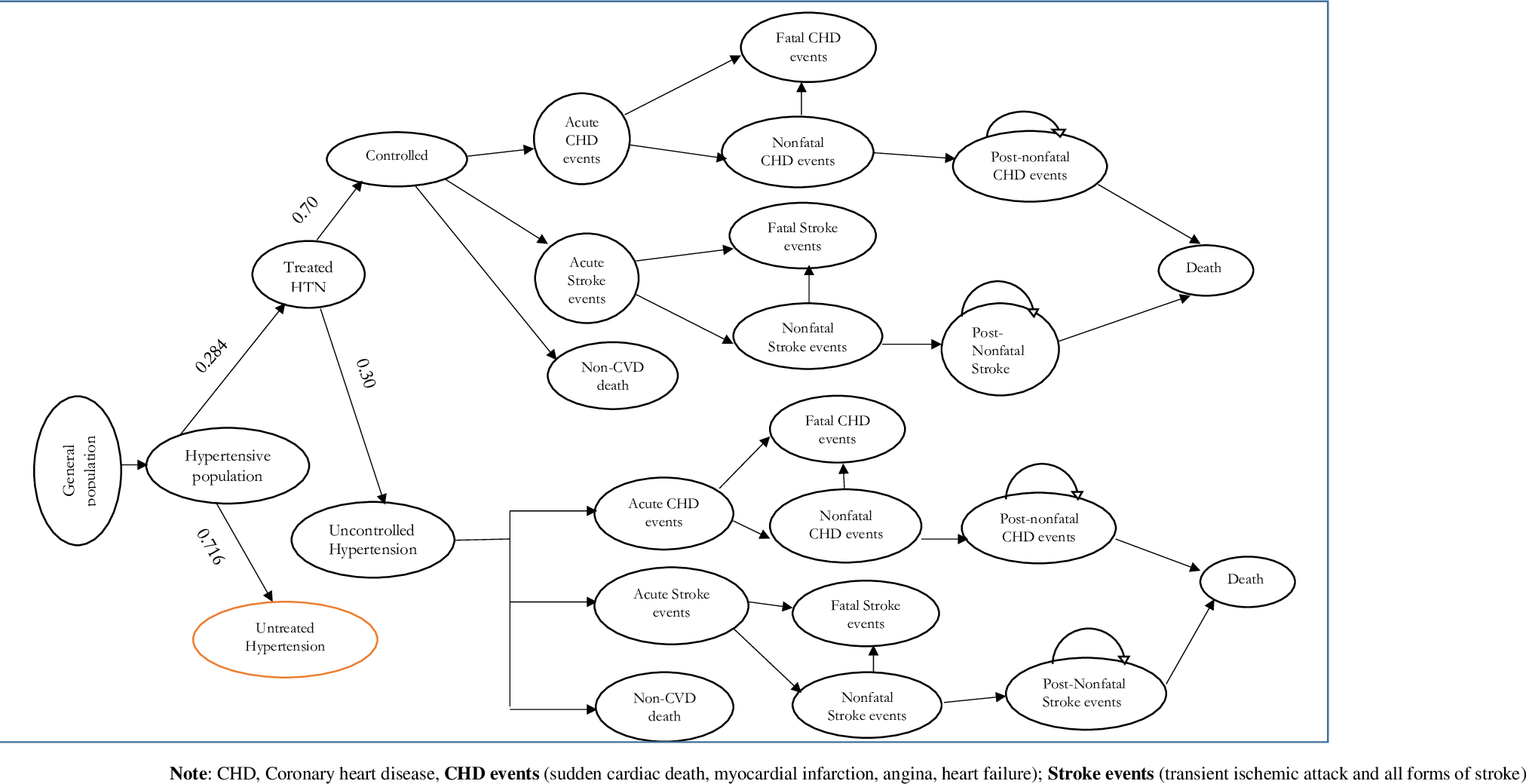

Supplement: S1 Fig — (TIF) [file pone.0273439.s001.tif]

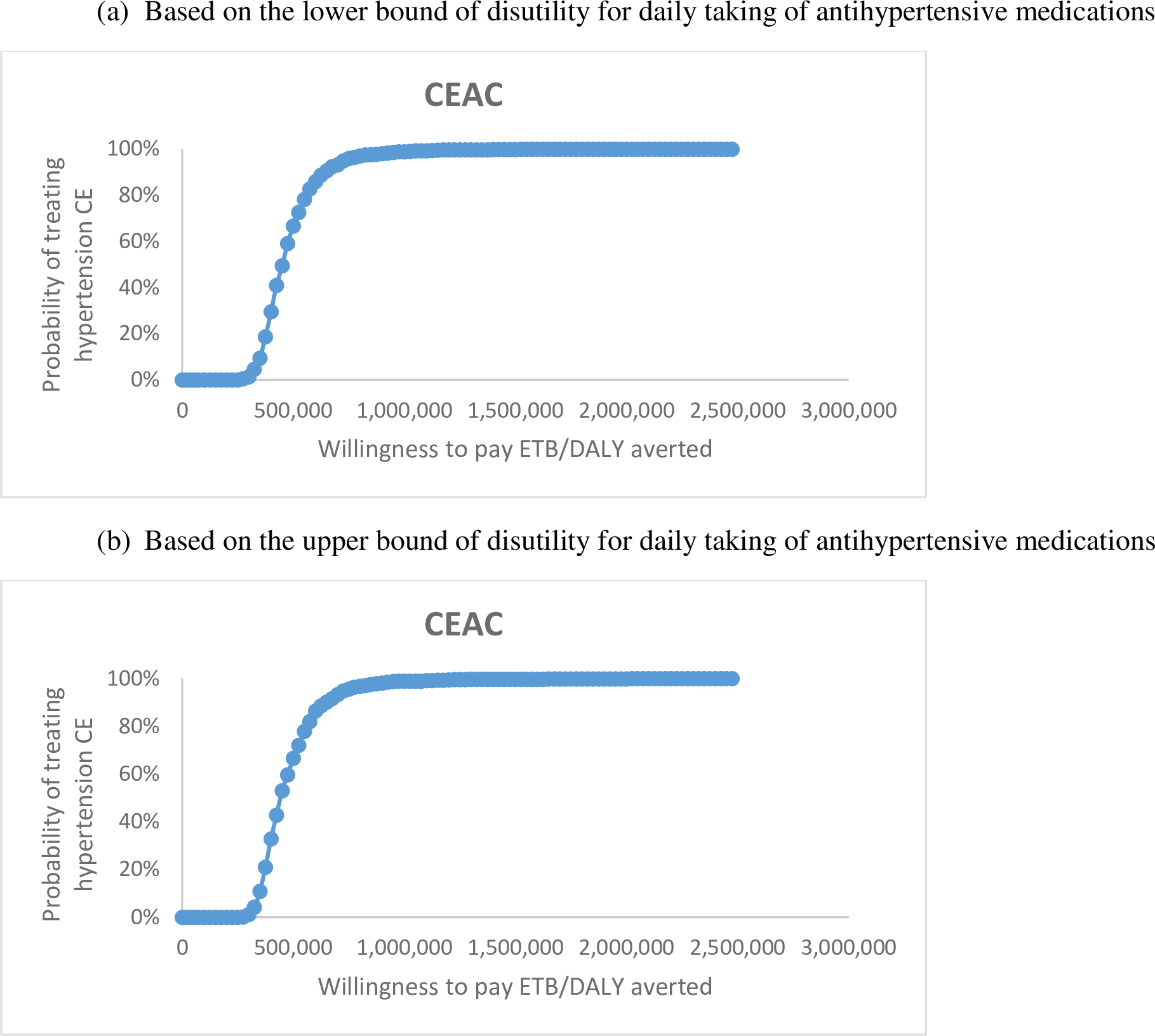

Supplement: S2 Fig — (TIF) [file pone.0273439.s002.tif]

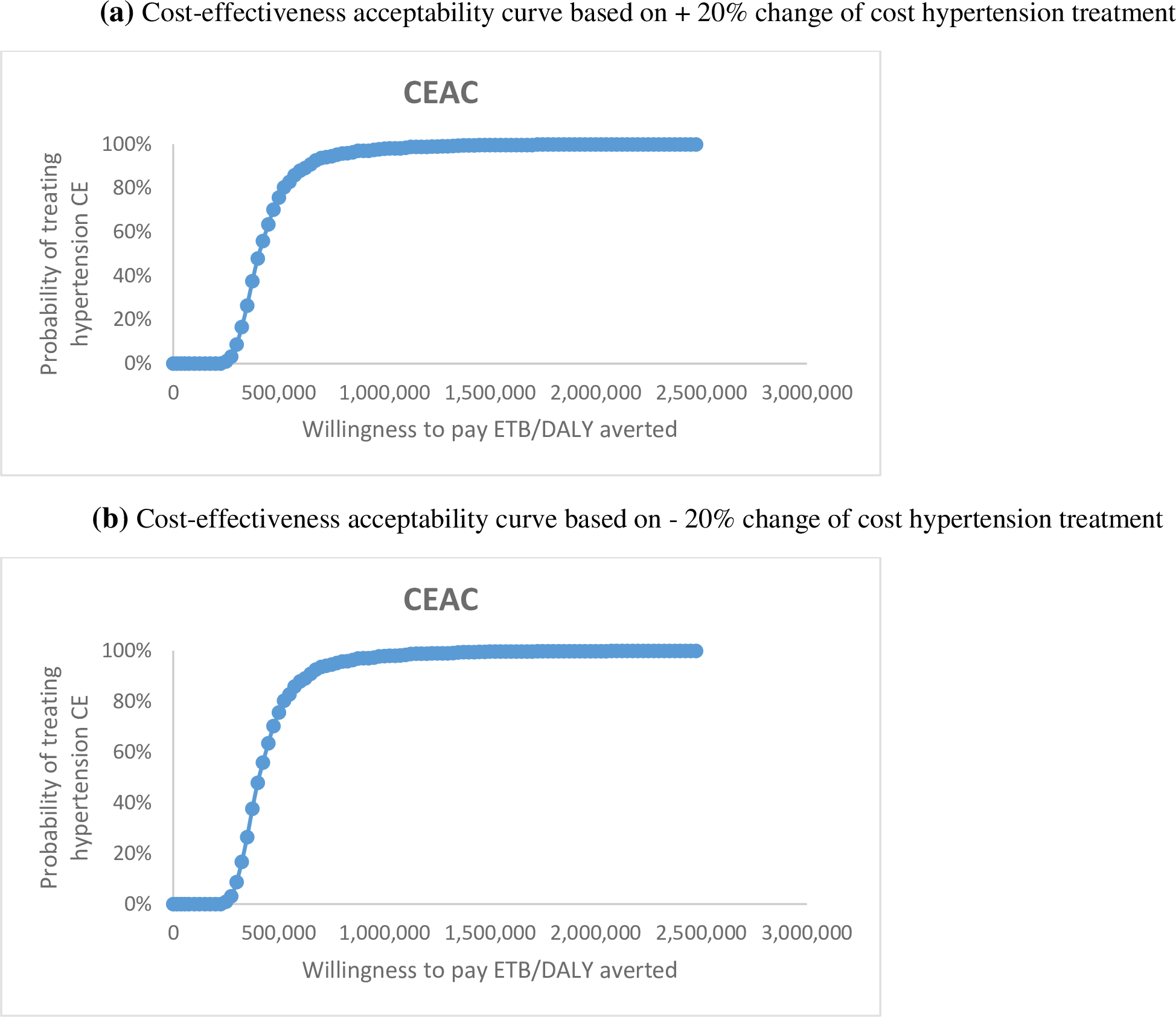

Supplement: S3 Fig — (TIF) [file pone.0273439.s003.tif]

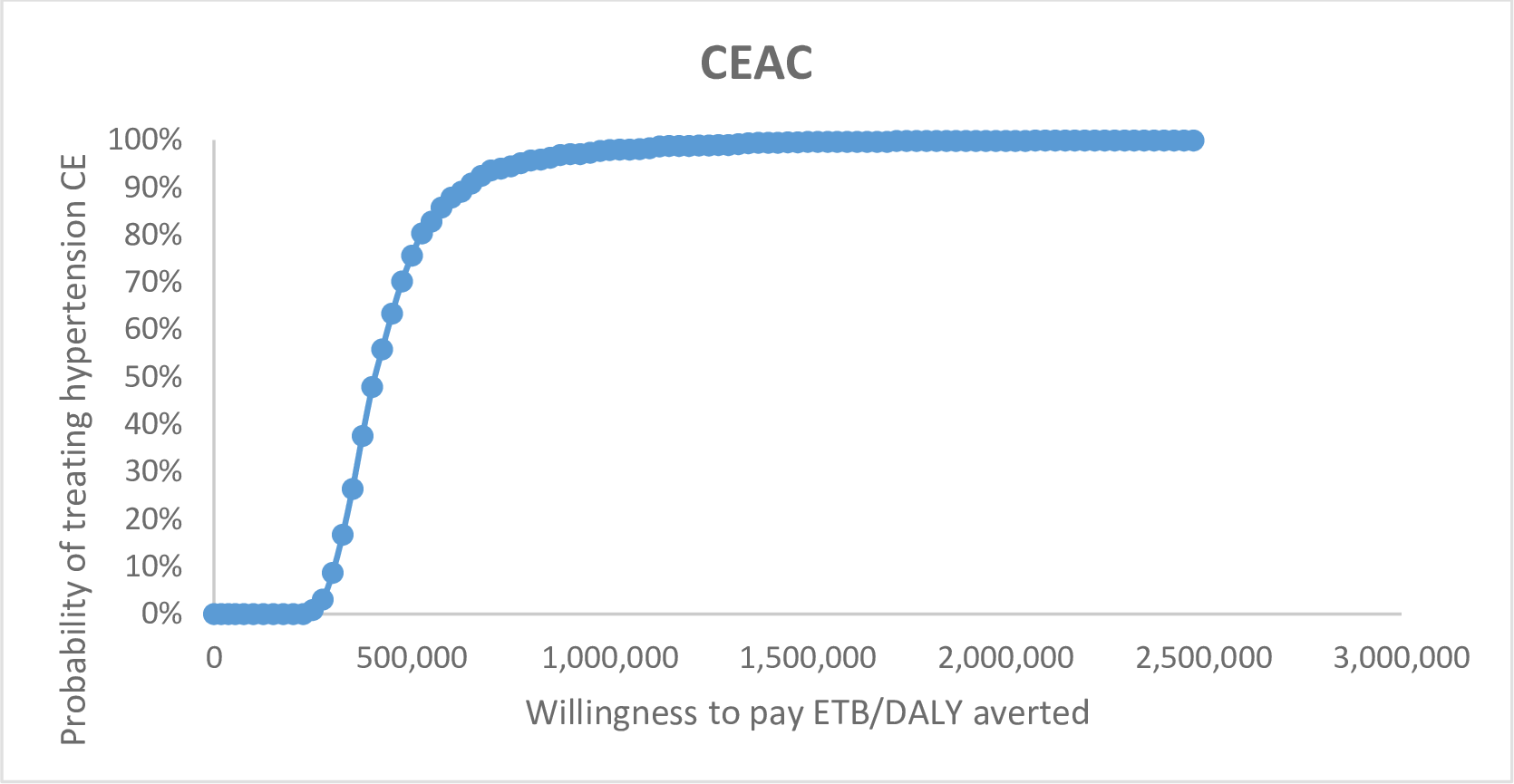

Supplement: S4 Fig — (TIF) [file pone.0273439.s004.tif]

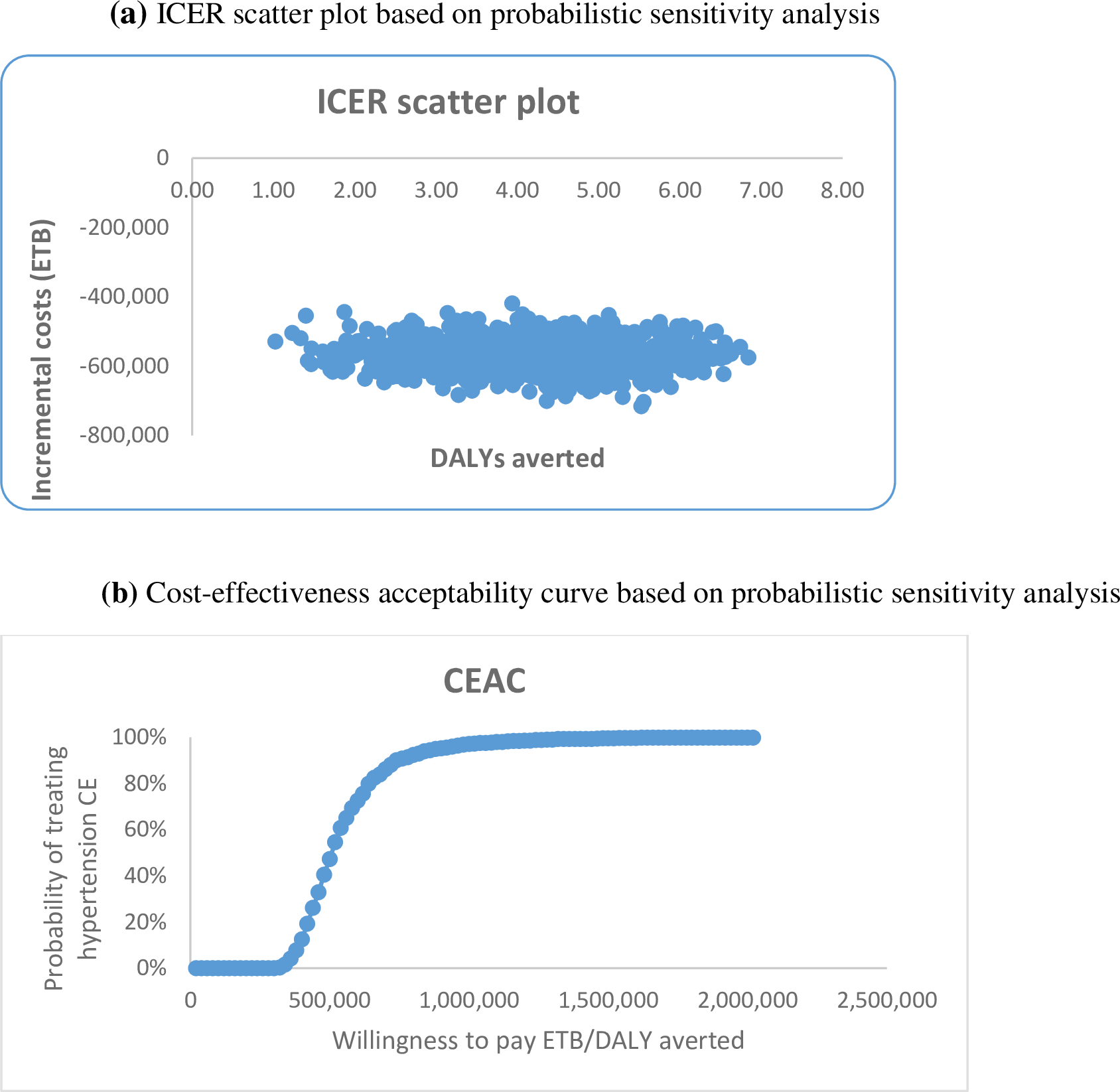

Supplement: S5 Fig — (TIF) [file pone.0273439.s005.tif]
