# Supplementary material for: Cost-effectiveness of hypertension therapy based on 2020 International Society of Hypertension guidelines in Ethiopia from a societal perspective
Source: PLoS One. 2022 Aug 29;17(8):e0273439. doi: 10.1371/journal.pone.0273439 (PMC9423649; doi:10.1371/journal.pone.0273439)
Supplement: S1 Table — (DOCX) [file pone.0273439.s006.docx]

**S1 Table.** Model Parameters and Probability of Transition between States

| Parameter | Data | Source |
| --- | --- | --- |
| Relative risk of hypertension treatment |  |  |
| Relative risk of CHD event on hypertension treatment | 0.683 (95% CI, 0.633–0.717) | [1-4] |
| Relative risk of a cerebrovascular event on hypertension treatment | 0.633 (95% CI, 0.526–0.717) | [5] |
| Relative risk of CHD event on normotensive men and women | 0.49 (95% CI 0.458–0.513) and 0.32 (0.292–0.342) | [6] |
| Transition probabilities to death |  |  |
| Probability of death eliminating major cardiovascular and cerebrovascular diseases | 0.07%–12.6%* |  |
| SMR after myocardial infarction | 2.68 (95% CI, 2.48–2.91) |  |
| SMR after unstable angina | 2.19 (95% CI, 2.05–2.33) |  |
| SMR after stable angina | 1.95 (95% CI, 1.65–2.31) |  |
| SMR after stroke | 2.27 (95% CI, 2.59–2.85) |  |
| SMR after transient ischemic attack | 1.4 (95% CI, 1.1–1.8) |  |
| Transition probabilities to the health states |  |  |
| Probability of CHD event if normotensive | 0.00%–0.26%* |  |
| Probability of CHD event if hypertensive | 0.00%–0.48%* |  |
| Probability of stroke event if normotensive | 0.00%–0.13%* |  |
| Probability of stroke event if hypertensive | 0.00%–0.25%* |  |
| Quality-of-life weights (utilities) |  |  |
| Baseline utilities for healthy population (i.e., no cardiovascular event) | 0.79 (SE, 0.005)–0.90 (SE, 0.003)* |  |
| Death | 0 |  |
| Quality-of-life multipliers† |  |  |
| Myocardial infarction | 0.76 (SE, 0.018) |  |
| Unstable angina | 0.77 (SE, 0.038) |  |
| Stable angina | 0.81 (SE, 0.038) |  |
| Stroke | 0.63 (SE, 0.04) |  |
| On hypertension treatment | 0.994 |  |
| Cost of diagnosis |  |  |

CHD, coronary heart disease; SMR, standardized mortality ratio. *Age and sex dependent †Applied multiplicatively to general population age- and sex-dependent utilities; CHD= Angina pectoris, coronary insufficiency, myocardial infarction, or coronary death.
