# Supplementary material for: Cost-effectiveness of hypertension therapy based on 2020 International Society of Hypertension guidelines in Ethiopia from a societal perspective
Source: PLoS One. 2022 Aug 29;17(8):e0273439. doi: 10.1371/journal.pone.0273439 (PMC9423649; doi:10.1371/journal.pone.0273439)
Supplement: S2 Table — (DOCX) [file pone.0273439.s007.docx]

**S2 Table.** Simulation input parameters and Disability weights for hypertension and related complications the Global Burden of Disease 2013 study and WHO Global Health Estimates

| **Input parameter** | **Value** | **Source** |
| --- | --- | --- |
| Non-CVD death rate | 0.005–0.176 (Age- and sex specific)# | Calculated from WHO lifetables and GBD 2017 [7] |
| Probability of first-time cardiovascular disease (CVD) event | Individual risk characteristic specific | Obtained from the Globorisk Office Calculator standardized for India |
| **Acute CVD events** |  |  |
| MI |  |  |
| Probability of MI if CVD event occurs | 37.6– 66.7% (Age- and sex specific)# | Calculated based on GBD 2017 [7] |
| 30-day fatality | 0.01–0.13 (Age- and sex– specific)# | Calibrated based on findings of Huffman et al. 2018 [8] |
| Re-infarction (in 30 days) | 0.0120 (0.0099–0·0141)ψ | ACS QUIK Study by Huffman et al. 2018 [8] |
| Acute Stroke (in 30 days) | 0.0060 (0.0045–0.0075)ψ | ACS QUIK Study by Huffman et al. 2018 [8] |
| Stroke |  |  |
| Probability of Stroke if CVD event occurs | 33.2–62.3% (Age- and sex specific)# | Calculated based on GBD 2017 [7] And  Jushua D. Bundry et al [9] |
| 30-day fatality | 0.12, 0.13 (Sex-specific)# | Calibrated based on a multi-site study by Pandian and Sudhan 2013 [30] |
| Repeat Stroke (in 30 days) | 0.15 (0.1–0.2)ψ | Petty et al. 1998 [10] |
| Sudden cardiac death | 0.10 per 100 patient-years (95% CI, 0.07–0.14) in a cohort of 33 of 3242 untreated hypertensive patients without evidence of coronary or cerebrovascular HD at entry and followed up for an average of 10.3 years | Heart disease and stroke statistics 2021 update |
| **Heart failure** |  |  |
| Probability of AHF |  |  |
| 30-days fatality | 0.0945 | Obtained from the THESUS-HF registry (11) and Korean Acute Heart Failure Registry (KorAHF) [12,13] |
| Re-hospitalization | 0.0736 | Obtained from the THESUS-HF registry [11] |
| **Chronic events** |  |  |
| Monthly risk of mortality | 0.001–0.019 (Age- and sex- specific)# | Calibrated based on GBD 2017 [7] |
| Reinfarction | 0.079 (0.073–0.085)ψ | Based on Steg et al. 2007 [14] and derived by Lin et al. 2019 [15] |
| Acute Stroke | 0.014 (0.012–0.016)ψ | Based on Steg et al. 2007 [14] and derived by Lin et al. 2019 [15] Continue Or Stop post-Stroke Antihypertensives Collaborative Study (COSSACS) [16], BP reduction and secondary stroke prevention: systematic review [17] |
| Stroke |  |  |
| Monthly risk of mortality | 0.001–0.013 (Age- and sex specific)# | Calibrated based on GBD 2017 [7] Stroke Risk in Treated Hypertension Based on Home Blood Pressure: the Ohasama Study [18] |
| Acute MI | 0.043 (0.038–0.048)ψ | Based on Steg et al. 2007 [14] and derived by Lin et al. 2019 [15] |
| Acute Stroke | 0.037 (0.033–0.041)ψ | Based on Steg et al. 2007 [14] and derived by Lin et al. 2019 [15] |
| Relative risk of fatality for an individual with two or more CVD events | 1.5 | Smolina et al. 2012 [19] |
| **Heart failure** |  |  |
| **Incidence** |  | Bulter J.et al [20, 21], and Davis BRK. et. al [22] |
| 1 year mortality |  |  |
| Re-hospitalization |  | Moita B.eta al. 2019 [23] and [24] |
| **Effect of antihypertensive medication** |  |  |
| Medication protocol for an individual | Initial SBP-specific# | Based on Ethiopian NCD control guideline |
| IHD relative risk due to medication | 0.32–0.89 (Age- and initial SBP-specific)# | Based on findings by Law et al. 2009 [25] and Asayam Kei., 2017 [26] |
| Stroke relative risk due to medication | 0.20–0.89 (Age- and initial SBP-specific)# | Based on findings by Law et al. 2009 [25] |
| IHD relative risk if partially adherent | 0.66–0.95 (Age- and initial SBP-specific) | Calculated based on a linear relationship between adherence and efficacy as considered by Cherry et al. 2009 [27] |
| Stroke relative risk if partially adherent | 0.60–0.95 (Age- and initial SBP-specific) | Calculated based on a linear relationship between adherence and efficacy as considered by Cherry et al. 2009 [27] and Lisheng Liu, Zengwu Wang. et al [28] |
| **Costs** |  |  |
| Programmatic Cost of Intervention | $22.83 per individual per annum# | Calculated from National strategic plan for NCD prevention and control 2016 |
| **Antihypertensive treatment** |  |  |
| Antihypertensive medication (per individual per annum) in public sector | $0.88–17.90 (Drug and dosage specific)§ | Drug costs based on national Drug supply agency wholesale price and retail price of selected hospitals |
| Antihypertensive medication (per individual per annum) in private sector | $5.42–$125.14 (Drug and dosage specific)§ | Average cost of the type and dosage drug dispensed is based on the treatment protocol |
| Out-patient consultations (per visit) | $43.36 | Annual outpatient visit cost (12*WHO cost per outpatient visit inflated to 2021) WHO Choice [29] |
| One-time diagnostic tests | $2.27 | Based on Laboratory procedures and test price of Arba Minch General Hospital, 2021 |
| **Acute CVD care** |  |  |
| In-patient costs for MI | $1040 | WHO Choice [29] inflated to 2021 |
| In-patient costs for Stroke | $940 | WHO Choice [29] inflated to 2021 |
| Chronic CVD care |  |  |
| Secondary care medication in public sector (per individual per annum) | $92, $184 (Dosage-specific)§ | MSH-2015 International Drug Price Indicator inflated to 2021 [11] |
| Outpatient cost for IHD (per annum) | $45 | WHO Choice [30] inflated to 2019–20 |
| Outpatient cost for Stroke (per annum) | $67 | WHO Choice [30] inflated to 2021 |
| **Disability Weights** |  |  |
| Disutility due to daily medication | 0.049 (0.031–0.072) | GBD disability weights [31] |
| Acute Events |  |  |
| Myocardial Infarction | 0.432 (0.288–0.579) | GBD disability weights [31] |
| Stroke | 0.570 (0.377–0.707) | GBD disability weights [31] |
| Occurrence of second or later CVD event | 0.985 (0.992–0.989) | GBD disability weights and Lin et al. 2019 [15] |
| Chronic States |  |  |
| Ischemic Heart Disease | 0.08 (0.02–0.24) | GBD disability weights [31] |
| Stroke | 0.135 (0.01–0.437) | GBD disability weights [31] |
| Alive post 2+ CVD Events | 0.242 (0.11–0.437) | GBD disability weights [31] |
| **Disability weights of Health states** | **Disability weight Estimate** | **Source** |
| **Hypertension** |  | [32] |
| Treated | 0.246 |  |
| Untreated | 0.323 |  |
| Treated and controlled | 0.171 |  |
| **Myocardial Infarction (MI)** |  | [33] |
| Day 1-2 | 0·432 |  |
| Days 3-28 | 0·074 |  |
| **Angina Pectoris** |  |  |
| Mild | 0·033 |  |
| Moderate | 0·080 |  |
| Severe | 0·167 |  |
| **Heart failure** |  |  |
| Mild | 0·041 |  |
| Moderate | 0·072 |  |
| Severe | 0·179 |  |
| **Stroke** |  |  |
| Long-term consequences, mild | 0·019 |  |
| Long-term consequences, moderate | 0·070 |  |
| Long-term consequences, moderate, plus cognition problems | 0·316 |  |
| Long-term consequences severe | 0·552 |  |
| Long-term consequences, severe, plus cognition problems | 0·588 |  |
| Stroke: long-term consequences, severe | 0.552 |  |
| Stroke: long-term consequences, severe plus cognition problems | 0.588 |  |
| **Diabetes, digestive, and genitourinary disease** |  |  |
| Diabetic neuropathy | 0.133 | [34] |
| Chronic kidney disease (stage IV) | 0.104 |  |
| End-stage renal disease: with kidney transplant | 0.024 |  |
| End-stage renal disease: on dialysis | 0.571 |  |
| Decompensated cirrhosis of the liver | 0.178 |  |
| Average inflation rate Ethiopia | 16.58% | <https://take-profit.org/en/statistics/inflation-rate/ethiopia/> |
| Average inflation rate foreign | 2.02% |  |
| Percentage change | 24.6% |  |
| Exchange rate July 2021 (1USD) | 43.5 ETB |  |
