# Supplementary material for: Cost-effectiveness of hypertension therapy based on 2020 International Society of Hypertension guidelines in Ethiopia from a societal perspective
Source: PLoS One. 2022 Aug 29;17(8):e0273439. doi: 10.1371/journal.pone.0273439 (PMC9423649; doi:10.1371/journal.pone.0273439)
Supplement: S3 Table — (DOCX) [file pone.0273439.s008.docx]

**S3 Table.** World health organization (WHO); Ethiopian life table 2019

| **Ethiopian life table** |  | **2019** | **2019** | **2019** |
| --- | --- | --- | --- | --- |
| Indicator | Age Group | Both sexes | Male | Female |
| Age-specific death rate | <1 year | 0.037508 | 0.042769 | 0.032082 |
| Age-specific death rate | 1-4 years | 0.003679 | 0.003846 | 0.003507 |
| Age-specific death rate | 5-9 years | 0.001152 | 0.001204 | 0.001098 |
| Age-specific death rate | 10-14 years | 0.00101 | 0.001151 | 0.000867 |
| Age-specific death rate | 15-19 years | 0.00098 | 0.001158 | 0.000799 |
| Age-specific death rate | 20-24 years | 0.00126 | 0.001498 | 0.001018 |
| Age-specific death rate | 25-29 years | 0.001539 | 0.001754 | 0.00132 |
| Age-specific death rate | 30-34 years | 0.001968 | 0.00214 | 0.001797 |
| Age-specific death rate | 35-39 years | 0.002712 | 0.002859 | 0.002569 |
| Age-specific death rate | 40-44 years | 0.003927 | 0.004216 | 0.003642 |
| Age-specific death rate | 45-49 years | 0.005558 | 0.006074 | 0.005035 |
| Age-specific death rate | 50-54 years | 0.0081 | 0.0093 | 0.006984 |
| Age-specific death rate | 55-59 years | 0.01162 | 0.013686 | 0.009814 |
| Age-specific death rate | 60-64 years | 0.018089 | 0.021376 | 0.015263 |
| Age-specific death rate | 65-69 years | 0.027401 | 0.031906 | 0.023409 |
| Age-specific death rate | 70-74 years | 0.044069 | 0.049938 | 0.039168 |
| Age-specific death rate | 75-79 years | 0.069384 | 0.076372 | 0.063365 |
| Age-specific death rate | 80-84 years | 0.108429 | 0.116529 | 0.101626 |
| Age-specific death rate | 85+ years | 0.183439 | 0.198188 | 0.171774 |
| Probability of dying | <1 year | 0.036549 | 0.041526 | 0.031377 |
| Probability of dying | 1-4 years | 0.014586 | 0.015243 | 0.01391 |
| Probability of dying | 5-9 years | 0.005742 | 0.006003 | 0.005475 |
| Probability of dying | 10-14 years | 0.005039 | 0.00574 | 0.004324 |
| Probability of dying | 15-19 years | 0.004888 | 0.005772 | 0.003988 |
| Probability of dying | 20-24 years | 0.006282 | 0.007463 | 0.005075 |
| Probability of dying | 25-29 years | 0.007664 | 0.008732 | 0.006577 |
| Probability of dying | 30-34 years | 0.009791 | 0.010641 | 0.008944 |
| Probability of dying | 35-39 years | 0.013469 | 0.014196 | 0.012765 |
| Probability of dying | 40-44 years | 0.019446 | 0.020859 | 0.018043 |
| Probability of dying | 45-49 years | 0.027411 | 0.029915 | 0.024863 |
| Probability of dying | 50-54 years | 0.039695 | 0.045441 | 0.034321 |
| Probability of dying | 55-59 years | 0.056457 | 0.066165 | 0.047896 |
| Probability of dying | 60-64 years | 0.086534 | 0.10146 | 0.07351 |
| Probability of dying | 65-69 years | 0.128224 | 0.147744 | 0.110574 |
| Probability of dying | 70-74 years | 0.198476 | 0.221978 | 0.178375 |
| Probability of dying | 75-79 years | 0.29564 | 0.32064 | 0.273501 |
| Probability of dying | 80-84 years | 0.426526 | 0.4512 | 0.405187 |
| Probability of dying | 85+ years | 1 | 1 | 1 |
| Expectation of life at age | <1 year | 68.6994 | 66.89559 | 70.52014 |
| Expectation of life at age | 1-4 years | 70.29415 | 68.78082 | 71.79484 |
| Expectation of life at age | 5-9 years | 67.31097 | 65.82069 | 68.78499 |
| Expectation of life at age | 10-14 years | 62.68527 | 61.20309 | 64.14988 |
| Expectation of life at age | 15-19 years | 57.99008 | 56.54196 | 59.41759 |
| Expectation of life at age | 20-24 years | 53.26265 | 51.85569 | 54.64547 |
| Expectation of life at age | 25-29 years | 48.58357 | 47.22683 | 49.91144 |
| Expectation of life at age | 30-34 years | 43.93948 | 42.62082 | 45.22532 |
| Expectation of life at age | 35-39 years | 39.34922 | 38.05232 | 40.61091 |
| Expectation of life at age | 40-44 years | 34.85234 | 33.56427 | 36.10368 |
| Expectation of life at age | 45-49 years | 30.49394 | 29.22605 | 31.72114 |
| Expectation of life at age | 50-54 years | 26.28291 | 25.05021 | 27.4662 |
| Expectation of life at age | 55-59 years | 22.26599 | 21.12371 | 23.35352 |
| Expectation of life at age | 60-64 years | 18.4487 | 17.44324 | 19.40257 |
| Expectation of life at age | 65-69 years | 14.95955 | 14.13058 | 15.74365 |
| Expectation of life at age | 70-74 years | 11.79213 | 11.14682 | 12.39011 |
| Expectation of life at age | 75-79 years | 9.09309 | 8.613848 | 9.53725 |
| Expectation of life at age | 80-84 years | 6.860393 | 6.499428 | 7.186523 |
| Expectation of life at age | 85+ years | 5.103469 | 4.787587 | 5.37898 |

**Source**: Life tables: Life tables by country Ethiopia <https://apps.who.int/gho/data/view.searo.60550?lang=en>. Accessed on 18-February 2022
