# Supplementary material for: Cost-effectiveness of hypertension therapy based on 2020 International Society of Hypertension guidelines in Ethiopia from a societal perspective
Source: PLoS One. 2022 Aug 29;17(8):e0273439. doi: 10.1371/journal.pone.0273439 (PMC9423649; doi:10.1371/journal.pone.0273439)
Supplement: S4 Table — (DOCX) [file pone.0273439.s009.docx]

**S4 Table.** Estimated working age population and percent distribution of women and men age 15-64 by employment status, according to, Ethiopia DHS 2016 and national STEPS survey and World population Prospect, 2020.

| Age | | Employed in the 12 months preceding the survey | | Data source |
| --- | --- | --- | --- | --- |
|  |  | Women (%) | Men (%) |  |
|  |  | Currently employed | Currently employed |  |
| 15-19 | | 24.3 | 68.6 | EDHS, 2016 [35] |
| 20-24 | | 31.0 | 84.4 |  |
| 25-29 | | 36.8 | 94.9 |  |
| 30-34 | | 39.9 | 96.6 |  |
| 35-39 | | 36.8 | 97.1 |  |
| 40-44 | | 36.7 | 96.4 |  |
| 45-49 | | 32.8 | 96.6 |  |
| 50-54 | | 7.8 | 7.8 | STEPS Survey, 2015 [36] |
| 55-59 | | 7.8 | 7.8 |  |
| 60-64 | | 5.4 | 5.4 |  |
| Estimated working age Population Ethiopia for year 2020 | | | |  |
| Age Group | Males | Females | Age Group Population | Data source |
| 15-19 | 5,572,330 | 5,464,174 | 11,036,504 | World population prospect 2020 estimate, years [37] |
| 20-24 | 5,930,683 | 5,816,173 | 11,746,856 |  |
| 25-29 | 4,889,739 | 4,802,450 | 9,692,189 |  |
| 30-34 | 3,761,349 | 3,757,544 | 7,518,893 |  |
| 35-39 | 3,091,148 | 3,182,837 | 6,273,985 |  |
| 40-44 | 2,445,523 | 2,488,422 | 4,933,945 |  |
| 45-49 | 2,071,480 | 2,033,228 | 4,104,708 |  |
| 50-54 | 1,567,789 | 1,660,957 | 3,228,746 |  |
| 55-59 | 1,159,002 | 1,316,318 | 2,475,320 |  |
| 60-64 | 946,594 | 1,109,670 | 2,056,264 |  |
