# Supplementary material for: Cost-effectiveness of hypertension therapy based on 2020 International Society of Hypertension guidelines in Ethiopia from a societal perspective
Source: PLoS One. 2022 Aug 29;17(8):e0273439. doi: 10.1371/journal.pone.0273439 (PMC9423649; doi:10.1371/journal.pone.0273439)
Supplement: S5 Table — (DOCX) [file pone.0273439.s010.docx]

**S5 Table.** Ethiopian population 2020 estimate and prevalence of hypertension in Ethiopia

| Age structure | Male | Female | Total | Estimated prevalence of hypertension (prevalence + incidence) | Population with hypertension | Source |
| --- | --- | --- | --- | --- | --- | --- |
| **Prevalence of hypertension** | | | | | | [36,38,39] |
| 0-14 years | 21,657,152 | 21,381,628 | 43,038,780 | NA |  |  |
| 15-24 years | 10,506,144 | 10,542,128 | 21,048,272 | 20.47 | 4,308,581 |  |
| 25-54 years | 17,720,540 | 17,867,298 | 35,587,838 | 21.6 | 7,686,973 |  |
| 55-64 years | 2,350,606 | 2,433,319 | 4,783,925 | 22.5 | 1,076,383 |  |
| ≥ 65 years | 1,676,478 | 1,977,857 | 3,654,335 | 23.2 | 847,806 |  |
| Total | 53,910,920 | 54,202,230 | 108,113,150 |  | **13,919,743** |  |
