# Supplementary material for: Cost-effectiveness of hypertension therapy based on 2020 International Society of Hypertension guidelines in Ethiopia from a societal perspective
Source: PLoS One. 2022 Aug 29;17(8):e0273439. doi: 10.1371/journal.pone.0273439 (PMC9423649; doi:10.1371/journal.pone.0273439)
Supplement: S6 Table — (DOCX) [file pone.0273439.s011.docx]

**S6 Table.** Risk of death across age and gender covariate categories stratified for hypertension [40]

| Variables | Categories | Incidence of death (%) | | Relative risk in each category (CI) |
| --- | --- | --- | --- | --- |
|  |  | High BP group | Normal |  |
| Age | 20-29 | 1.68% | 0.54% | 3.11 (1.16-8.36) |
|  | 30-39 | 1.71% | 0.94% | 1.82 (1.04-3.19) |
|  | 40-49 | 2.43% | 1.88% | 1.29 (0.91-1.82) |
|  | 50-59 | 6.30% | 4.03% | 1.56 (1.28-1.91) |
|  | 60 and above | 19.32% | 15.9% | 1.21 (1.12-1.31) |
| Gender | Women | 8.71% | 1.1% | 3.31 (2.98-3.68) |
|  | Men | 15.47% | 4.62% | 3.34(3.02-3.70) |
