# Supplementary material for: Cost-effectiveness of hypertension therapy based on 2020 International Society of Hypertension guidelines in Ethiopia from a societal perspective
Source: PLoS One. 2022 Aug 29;17(8):e0273439. doi: 10.1371/journal.pone.0273439 (PMC9423649; doi:10.1371/journal.pone.0273439)
Supplement: S7 Table — (DOCX) [file pone.0273439.s012.docx]

**S7 Table**. Age-sex specific mortality [41]

| Death rate/1000 | Age range |  |  |
| --- | --- | --- | --- |
|  | 15-49 | 50-69 | ≥ 70 years |
| All case | 7614·0 (7496·5 to 7741·4) | 14 998·6 (14 827·9 to 15 170·8) | 27209·8 (26 97·2 to 27 441·9) |
| CVD | 1258·0 (1234·6 to 1284·7) | **5152·1 (5068·6 to 5233·7)** | 11335·1 (11173·0 to 11494·9) |
| IHD | 643·8 (628·9 to 661·2) | 2649·1 (2602·9 to 2699·1) | 5637·5 (5547·9 to 5786·4) |
| Stroke | 364·2 (354·9 to 375·0) | 1836·6 (1795·9 to 1879·2) | 3953·8 (3875·7 to 4067·2) |
| HHD | 43·8 (32·4 to 49·2) | 224·2 (172·2 to 242·8) | 657·7 (473·8 to 710·6) |
| **Non-CVD death** | 6356.0 (4365.55 to 4513.05) | 9845.1 (9948.25 to 10202.250 | 15884.7 (19074.6 to 19468.4) |
