# Supplementary material for: Cost-effectiveness of hypertension therapy based on 2020 International Society of Hypertension guidelines in Ethiopia from a societal perspective
Source: PLoS One. 2022 Aug 29;17(8):e0273439. doi: 10.1371/journal.pone.0273439 (PMC9423649; doi:10.1371/journal.pone.0273439)
Supplement: S8 Table — (DOCX) [file pone.0273439.s013.docx]

**S8 Table**. Percent distribution of Adult Mortality rates, among 15-49, Ethiopia DHS 2016.

| Variables | | Mortality rate | | Data source |
| --- | --- | --- | --- | --- |
|  |  | Women | Men |  |
| Age | 15-19 | 0.00222 | 0.00286 | [35, 40] |
|  | 20-24 | 0.00223 | 0.00319 |  |
|  | 25-29 | 0.002.32 | 0.00293 |  |
|  | 30-34 | 0.003.68 | 0.00397 |  |
|  | 35-39 | 0.00222 | 0.00411 |  |
|  | 40-44 | 0.00385 | 0.00584 |  |
|  | 45-49 | 0.00457 | 0.00360 |  |
|  | 50-54 | 0.00274 | 0.00354 |  |
|  | 55-59 | 0.00274 | 0.00354 |  |
|  | 60-64 | 0.00274 | 0.00354 |  |
|  | ≥ 65 years | 0.00274 | 0.00354 |  |
