# Supplementary material for: Cost-effectiveness of hypertension therapy based on 2020 International Society of Hypertension guidelines in Ethiopia from a societal perspective
Source: PLoS One. 2022 Aug 29;17(8):e0273439. doi: 10.1371/journal.pone.0273439 (PMC9423649; doi:10.1371/journal.pone.0273439)
Supplement: S9 Table — (DOCX) [file pone.0273439.s014.docx]

**S9 Table.** Annual mortality rate in the total population, those with hypertension by treatment and control status and those without hypertension in Ethiopia in 2021 by age group and sex based on literature review of systematic reviews and clinical trials

| Age group | Mortality rate in the total population | Mortality rate among people without hypertension | Mortality rate among people with treated and controlled hypertension | Mortality rate among people with treated but uncontrolled hypertension | Mortality rate among people with untreated hypertension | References |
| --- | --- | --- | --- | --- | --- | --- |
| **Women** |  |  |  |  |  |  |
| 15-19 | 0.000799 | 0.000799 | 0.000856 | 0.00336 | 0.002786 | Ko, Min Jung. et al. 2016 [42], Mende Sorato, et al, 2021 [9,43-45]. |
| 20-24 | 0.001018 | 0.001018 | 0.000856 | 0.00336 | 0.002786 |  |
| 25-29 | 0.00132 | 0.00132 | 0.000856 | 0.00336 | 0.002786 |  |
| 30-34 | 0.001797 | 0.001797 | 0.000856 | 0.00336 | 0.002786 |  |
| 35-39 | 0.002569 | 0.002569 | 0.000856 | 0.00336 | 0.002786 |  |
| 40-44 | 0.003642 | 0.003642 | 0.000856 | 0.00336 | 0.002786 |  |
| 45-49 | 0.005035 | 0.005035 | 0.000856 | 0.00336 | 0.002786 |  |
| 50-54 | 0.006984 | 0.006984 | 0.000856 | 0.00336 | 0.002786 |  |
| 55-59 | 0.009814 | 0.009814 | 0.000856 | 0.00336 | 0.002786 |  |
| 60 -64 | 0.015263 | 0.015263 | 0.000856 | 0.00336 | 0.002786 |  |
| **Men** |  |  |  |  |  |  |
| 15-19 | 0.001158 | 0.001158 | 0.00122 | 0.00173 | 0.00232 | Kuriakose A. et al. 2014 [40], EDHS, 2016 [35, 44-47] |
| 20-24 | 0.001498 | 0.001498 | 0.00122 | 0.00173 | 0.00232 |  |
| 25-29 | 0.001754 | 0.001754 | 0.00122 | 0.00173 | 0.00232 |  |
| 30-34 | 0.00214 | 0.00214 | 0.00122 | 0.00173 | 0.00232 |  |
| 35-39 | 0.002859 | 0.002859 | 0.00122 | 0.00173 | 0.00232 |  |
| 40-44 | 0.004216 | 0.004216 | 0.00122 | 0.00173 | 0.00232 |  |
| 45-49 | 0.006074 | 0.006074 | 0.00122 | 0.00173 | 0.00232 |  |
| 50-54 | 0.0093 | 0.0093 | 0.00311 | 0.00373 | 0.00232 |  |
| 55-59 | 0.013686 | 0.013686 | 0.00311 | 0.00373 | 0.00232 |  |
| 60-64 | 0.021376 | 0.021376 | 0.00311 | 0.00924 | 0.00232 |  |
