# Supplementary material for: Cost-effectiveness of hypertension therapy based on 2020 International Society of Hypertension guidelines in Ethiopia from a societal perspective
Source: PLoS One. 2022 Aug 29;17(8):e0273439. doi: 10.1371/journal.pone.0273439 (PMC9423649; doi:10.1371/journal.pone.0273439)
Supplement: S11 Table — (DOCX) [file pone.0273439.s016.docx]

**S11 Table.** Estimates of preventive effect of taking one or more blood pressure lowering drugs on coronary heart disease (CHD) events and stroke according to pretreatment diastolic blood pressure, age, number of drugs, and dose (as multiple of standard33)32 then the effect of this blood pressure reduction on disease risk [49].

| Pretreatment diastolic blood pressure (mm Hg) | Estimated reduction in diastolic blood pressure (mm Hg)* | Relative risk of CHD events by age (years) | | | | | Relative risk of stroke by age (years) | | | | |
| --- | --- | --- | --- | --- | --- | --- | --- | --- | --- | --- | --- |
|  |  | 40-49 | 50-59 | 60-69 | 70-79 | 80-89 | 40-49 | 50-59 | 60-69 | 70-79 | 80-89 |
| One drug half dose |  |  |  |  |  |  |  |  |  |  |  |
| 110 | 5.5 | 0.66 | 0.70 | 0.73 | 0.77 | 0.82 | 0.56 | 0.55 | 0.61 | 0.67 | 0.78 |
| 105 | 5 | 0.68 | 0.72 | 0.75 | 0.79 | 0.84 | 0.59 | 0.58 | 0.63 | 0.69 | 0.79 |
| 100 | 4.6 | 0.71 | 0.74 | 0.77 | 0.80 | 0.85 | 0.62 | 0.61 | 0.66 | 0.71 | 0.81 |
| 95 | 4.1 | 0.73 | 0.76 | 0.79 | 0.82 | 0.86 | 0.65 | 0.64 | 0.68 | 0.74 | 0.83 |
| 90 | 3.7 | 0.76 | 0.79 | 0.81 | 0.84 | 0.88 | 0.68 | 0.67 | 0.71 | 0.76 | 0.84 |
| 85 | 3.3 | 0.78 | 0.81 | 0.83 | 0.86 | 0.89 | 0.71 | 0.70 | 0.74 | 0.79 | 0.86 |
| 80 | 2.8 | 0.81 | 0.83 | 0.85 | 0.87 | 0.90 | 0.74 | 0.74 | 0.77 | 0.81 | 0.88 |
| 75 | 2.4 | 0.84 | 0.86 | 0.87 | 0.89 | 0.92 | 0.78 | 0.77 | 0.80 | 0.84 | 0.90 |
| One drug std dose |  |  |  |  |  |  |  |  |  |  |  |
| 110 | 6.9 | 0.59 | 0.64 | 0.67 | 0.72 | 0.78 | 0.48 | 0.47 | 0.53 | 0.60 | 0.73 |
| 105 | 6.4 | 0.62 | 0.66 | 0.69 | 0.74 | 0.80 | 0.51 | 0.50 | 0.56 | 0.63 | 0.74 |
| 100 | 5.8 | 0.64 | 0.68 | 0.71 | 0.76 | 0.81 | 0.54 | 0.53 | 0.59 | 0.65 | 0.76 |
| 95 | 5.3 | 0.67 | 0.71 | 0.74 | 0.78 | 0.83 | 0.57 | 0.57 | 0.62 | 0.68 | 0.78 |
| 90 | 4.7 | 0.70 | 0.73 | 0.76 | 0.80 | 0.84 | 0.61 | 0.60 | 0.65 | 0.71 | 0.80 |
| 85 | 4.2 | 0.73 | 0.76 | 0.78 | 0.82 | 0.86 | 0.64 | 0.64 | 0.68 | 0.74 | 0.82 |
| 80 | 3.6 | 0.76 | 0.79 | 0.81 | 0.84 | 0.88 | 0.68 | 0.68 | 0.72 | 0.77 | 0.85 |
| 75 | 3.1 | 0.79 | 0.82 | 0.84 | 0.86 | 0.90 | 0.72 | 0.72 | 0.75 | 0.80 | 0.87 |
| Two drug half dose |  |  |  |  |  |  |  |  |  |  |  |
| 110 | 10.8 | 0.44 | 0.49 | 0.53 | 0.60 | 0.68 | 0.32 | 0.31 | 0.37 | 0.45 | 0.61 |
| 105 | 9.9 | 0.47 | 0.52 | 0.56 | 0.62 | 0.70 | 0.35 | 0.34 | 0.40 | 0.48 | 0.63 |
| 100 | 9.1 | 0.50 | 0.55 | 0.59 | 0.65 | 0.72 | 0.39 | 0.38 | 0.44 | 0.51 | 0.66 |
| 95 | 8.2 | 0.54 | 0.59 | 0.62 | 0.68 | 0.75 | 0.42 | 0.41 | 0.47 | 0.55 | 0.69 |
| 90 | 7.3 | 0.58 | 0.62 | 0.65 | 0.71 | 0.77 | 0.46 | 0.45 | 0.51 | 0.59 | 0.71 |
| 85 | 6.4 | 0.62 | 0.66 | 0.69 | 0.74 | 0.80 | 0.51 | 0.50 | 0.56 | 0.62 | 0.74 |
| 80 | 5.5 | 0.66 | 0.70 | 0.73 | 0.77 | 0.82 | 0.56 | 0.55 | 0.60 | 0.67 | 0.77 |
| 75 | 4.7 | 0.70 | 0.74 | 0.76 | 0.80 | 0.85 | 0.61 | 0.60 | 0.65 | 0.71 | 0.81 |
| two drug std dose |  |  |  |  |  |  |  |  |  |  |  |
| 110 | 13.1 | 0.37 | 0.42 | 0.49 | 0.53 | 0.63 | 0.25 | 0.24 | 0.30 | 0.38 | 0.55 |
| 105 | 12.1 | 0.40 | 0.45 | 0.50 | 0.56 | 0.65 | 0.28 | 0.27 | 0.33 | 0.41 | 0.57 |
| 100 | 11.0 | 0.44 | 0.49 | 0.53 | 0.59 | 0.67 | 0.31 | 0.30 | 0.36 | 0.45 | 0.60 |
| 95 | 11.0 | 0.44 | 0.49 | 0.53 | 0.59 | 0.67 | 0.31 | 0.30 | 0.36 | 0.45 | 0.60 |
| 90 | 10.0 | 0.47 | 0.52 | 0.56 | 0.62 | 0.70 | 0.35 | 0.34 | 0.40 | 0.48 | 0.63 |
| 85 | 8.9 | 0.51 | 0.56 | 0.60 | 0.65 | 0.73 | 0.39 | 0.38 | 0.44 | 0.52 | 0.66 |
| 80 | 6.9 | 0.60 | 0.64 | 0.67 | 0.72 | 0.78 | 0.49 | 0.48 | 0.53 | 0.60 | 0.73 |
| 75 | 5.8 | 0.64 | 0.68 | 0.71 | 0.76 | 0.81 | 0.54 | 0.53 | 0.59 | 0.65 | 0.76 |
| Three drug half dose |  |  |  |  |  |  |  |  |  |  |  |
| 110 | 16.0 | 0.30 | 0.35 | 0.40 | 0.47 | 0.57 | 0.19 | 0.18 | 0.23 | 0.31 | 0.48 |
| 105 | 14.7 | 0.33 | 0.38 | 0.43 | 0.50 | 0.59 | 0.21 | 0.21 | 0.26 | 0.34 | 0.51 |
| 100 | 13.3 | 0.37 | 0.42 | 0.46 | 0.53 | 0.62 | 0.25 | 0.24 | 0.29 | 0.38 | 0.54 |
| 95 | 12.0 | 0.40 | 0.46 | 0.50 | 0.56 | 0.65 | 0.28 | 0.27 | 0.33 | 0.41 | 0.57 |
| 90 | 10.7 | 0.45 | 0.50 | 0.54 | 0.60 | 0.68 | 0.33 | 0.32 | 0.38 | 0.46 | 0.61 |
| 85 | 9.4 | 0.49 | 0.54 | 0.58 | 0.64 | 0.72 | 0.37 | 0.36 | 0.42 | 0.50 | 0.65 |
| 80 | 8.1 | 0.54 | 0.59 | 0.63 | 0.68 | 0.75 | 0.43 | 0.42 | 0.48 | 0.55 | 0.69 |
| 75 | 6.7 | 0.60 | 0.64 | 0.68 | 0.72 | 0.79 | 0.49 | 0.48 | 0.54 | 0.61 | 0.73 |
| Three drug std. dose |  |  |  |  |  |  |  |  |  |  |  |
| 110 | 18.6 | 0.25 | 0.30 | 0.34 | 0.41 | 0.52 | 0.14 | 0.13 | 0.18 | 0.26 | 0.42 |
| 105 | 17.1 | 0.27 | 0.33 | 0.37 | 0.44 | 0.54 | 0.17 | 0.16 | 0.21 | 0.28 | 0.45 |
| 100 | 15.6 | 0.31 | 0.36 | 0.40 | 0.47 | 0.57 | 0.19 | 0.19 | 0.24 | 0.32 | 0.49 |
| 95 | 14.2 | 0.34 | 0.40 | 0.44 | 0.51 | 0.60 | 0.23 | 0.22 | 0.27 | 0.35 | 0.52 |
| 90 | 12.7 | 0.38 | 0.44 | 0.48 | 0.55 | 0.64 | 0.26 | 0.25 | 0.31 | 0.39 | 0.56 |
| 85 | 11.2 | 0.43 | 0.48 | 0.52 | 0.59 | 0.67 | 0.31 | 0.30 | 0.36 | 0.44 | 0.60 |
| 80 | 9.7 | 0.48 | 0.53 | 0.57 | 0.63 | 0.71 | 0.36 | 0.35 | 0.41 | 0.49 | 0.64 |
| 75 | 8.3 | 0.54 | 0.58 | 0.62 | 0.67 | 0.74 | 0.42 | 0.41 | 0.47 | 0.55 | 0.68 |
