# Supplementary material for: Cost-effectiveness of hypertension therapy based on 2020 International Society of Hypertension guidelines in Ethiopia from a societal perspective
Source: PLoS One. 2022 Aug 29;17(8):e0273439. doi: 10.1371/journal.pone.0273439 (PMC9423649; doi:10.1371/journal.pone.0273439)
Supplement: S1 File — (PDF) [file pone.0273439.s017.pdf]

9. Annexes

Annex I: Consent Letter

Title of the research: Evaluation of hypertension treatment effectiveness, drug prescribing pattern, and economic burden of hypertension and cost-effectiveness of hypertension treatment at selected Hospitals in Southern Ethiopia

My name is ------------------------------ Address --

I am working as a data collector for study on evaluation of hypertension treatment effectiveness, drug prescribing pattern, and economic burden of hypertension and cost-effectiveness of hypertension treatment at selected Hospitals in Southern Ethiopia. The objective of the study is *to evaluate of hypertension treatment effectiveness, drug prescribing pattern, and economic burden of hypertension and cost-effectiveness of hypertension treatment at selected Hospitals in Southern Ethiopia*

During the interview you will be asked some short questions about your Socio-demographic characteristics and other information's regarding hypertension management. Your answers will be recorded on a survey questionnaire. No personal identifiers will be recorded to the interview. All the data obtained will be kept strictly confidential by using only code numbers. Your participation in the study is upon purely voluntary basis. What we learn from this study will be used to generate information necessary for the planning to improve, redesign and scale up hypertension management practice in our country particularly in our hospitals. The interview will be conducted in private and will take 20-30 minutes. During the interview period, if you feel inconvenient, you can interrupt and clarify inconvenience, appoint to other time or even withdraw any time after you get involved in the study. Your honest and genuine participation in responding to the questions prepared is very important & highly appreciated.

If you agree to participate in this study I will interview you.

The purpose of the study and confidentiality of procedures has been explained to me and I on my own consent: a) Agree _____ b) Disagree_____

Interviewer name ______________________ Signature ______________________

Checked by supervisor: Name__________________ Signature_______ Date_______

Annex II: Questionnaire

I. Patient interview Questionnaire

Patient Card Number _______________ Code Number ________________Date ________________		
**Instruction to the interviewer:** Encircle the number where choices are given and fill blank space for open ended questions during patient interview, and chart review appropriately. **1a. Socio demographic data**		
1.	Sex	1. Male 2. Female
2.	Age	________________ years
3.	Religion	1. Orthodox 2. Muslim 3.Protestant 4. Catholic 5. Others-----------
4.	Ethnicity	1. Gamo 2. Wolyta 3. Gofa 4. Konso 5. Amhara 6. Other _____
5.	Marital status	1. Married 3. Widowed 2. Divorced 4. Single/ never married
6.	Monthly Income	In Birr __________________
7.	Level of education	1. Illiterate 2. If Literate(Yrs. completed)______________
8.	Occupation/ employment	1. Employed 2. Merchant 3. Unemployed 4. Farmer 5. Others specify: _______________
**1b. Disease-related characteristics data**		
9.	Disease duration since diagnosis	____________ months/ years
10.	Family history of (CVDs)	1. 1st Degree relative 2. 2nd Degree relative 3. None
11.	Family history of diabetes	1. 1st Degree relative 2. 2nd Degree relative 3. None
12.	Do you have any disease seeking treatment/under treatment other than hypertension 1. Yes 2. No	
13.	If yes; **Which of the following physical and psychological problems do you have?** You may encircle more than one A. Myocardial infarction B. Heart failure C. Stroke D. Diabetes E. Thyroid disease F. Kidney disease G. Liver disease H. Asthma I. Erectile Dysfunction J. Others Specify:_____________________	
14.	Smoking status	1. Never 2. Passive Smoker 3. Current Smoker (__________# of cigarettes)
15.	Alcohol drinking status	1. Never 2. Current drinker (in past month how many times more than 2 drinks/day
16.	Physical activity	Type of work____________________<br>Means of travel__________________<br>Activities other than work________________
17.	Dietary factors	1. Number of fruits serving per day _______________<br>2. Number of vegetables serving per day ________________<br>3. number of saturated fats (butter, eggs, meat) per day _________<br>4. Amount of table salt added per servings _____________

1c. Patient Knowledge

The hypertension evaluation of lifestyle and management (HELM) Knowledge Scale	
**Instructions**: On the following page, you will be asked to respond to a number of True/False questions addressing your beliefs and knowledge about various aspects of heart disease. Please answer each by circling correct alternative. Very few people answer all these questions correctly just do the best you can. Feel free to circle 'Don't know' if you are unsure of an answer.	
**Item Content**	**Response**
1. A person is considered to have hypertension if either their systolic blood pressure is 140 or their diastolic is 90 or higher on two separate occasions.	True False
2. Most people can tell when their blood pressure is high because they feel bad.	True False
3. Uncontrolled hypertension can lead to:	A. Cancer B. Kidney failure C. High cholesterol
4. The most important cause of heart attacks is stress.	True False
5. Most of the cholesterol in an egg is in the white part of the egg.	True False
6. A healthy person's pulse should return to normal within 15 minutes after exercise.	True False
7. People who have diabetes are at higher risk of getting heart disease.	True False
8. Trans-fats are healthier for the heart than most other kinds of fats.	True False
9. Polyunsaturated fats are healthier for the heart than saturated fats.	True False
10. Smokers are more likely to die of lung cancer than heart disease.	True False
11. Which of the following increases your risk of having hypertension?	Weight lifting Drinking >2 cups of coffee a day Smoking a pack of cigarettes Gaining 15 pounds
12. People with hypertension do not need to take medicine if they exercise regularly	True False
13. Which of the following statements about taking blood pressure medicine is TRUE?	A. Should always be taken with food<br>B. Should be taken for life long<br>C. BP medicine works best if it is taken at bedtime<br>D. BP medicine should not be taken if a person drank alcohol that day
14. Most of the salt Americans eat is added with a salt shaker.	True False
15. Which one of the following changes is the most likely to lower blood pressure?	A. Lose 10 pounds B. Stop drinking alcohol C. Switch to decaffeinated coffee D. Switch to sea salt
16. Which one of the following changes to your diet is most likely to lower blood pressure?	A. Eat more fruits, vegetables B. Drink herbal tea instead of coffee C. Eat meat D. Replace saturated oils with unsaturated oil
17. Which one of the following statements about exercise and blood pressure is TRUE?	A. Normal weight persons will not benefit from exercise<br>B. Exercising for 30 minutes every day lowers blood pressure<br>C. Weight lifting should be avoided by people with hypertension<br>D. Routine daily work is sufficient to improve blood pressure
18. If someone is told that their goal blood pressure is 130/80 mmHg, when will he/she reach the goal BP?	A. He/she is at target now B. When the SBP is below 120 C. When the DBP is below 80 D. When SBP is below 120 and SBP is below 80
**Abbreviation**: HELM, hypertension evaluation of lifestyle and management. Correct responses are underlined.	

Ie. Nine-item shared decision making questionnaire (SDM-Q-9)

Health compliant/illness:	Hypertension			
Nine statements related to the decision-making in your consultation are listed below. For each statement please indicate how much you agree or disagree				
	Strongly disagree (0)	Somewh at disagree (1)	Somewhat agree (2)	Strongly agree (4)
1. My doctor made clear that a decision needs to be made				
2. My doctor wanted to know exactly how I want to be involved in making the decision				
3. My doctor told me that there are different options for treating hypertension				
4. my doctor precisely explained the advantages and disadvantages of treatment options				
5. My doctor helped me understand all the information				
6. My doctor asked me which treatment option I prefer				
7. My doctor and I thoroughly weighed the different treatment options				
8. My doctor and I selected a treatment option together				
9. My doctor and I reached an agreement on how to proceed				

Ie. Questions for the assessment of psychosocial risk factors Hypertension

Perceived Stress Scale: The questions in this scale ask you about your feelings and thoughts during the last month. Please indicate by circling how often you felt or thought a certain way

Perceived stress Scale (PSS)−10 item: In the last month:	**Score**				
	Never (0)	Almost never (1)	Sometimes (2)	Fairly often (3)	Very often (4)
1. How often have you been upset because of something that happened unexpectedly?					
2. How often have you felt that you were unable to control the important things in your life?					
3. How often have you felt nervous and “stressed”?					
4. How often have you felt confident about your ability to handle your personal problems?					
5. How often have you felt that things were going your way?					
6. How often have you found that you could not cope with all the things that you had to do?					
7. How often have you been able to control irritations in life?					
8. How often have you felt that you were on top of things?					
9. How often have you been angered because of things that were outside of your control?					
10. How often have you felt difficulties were piling up so high that you could not overcome them?					
**Total Score**					
**I. Hypertension related depression**					
**The following questions were about your feelings Over the last 2 weeks, how often have you been bothered by any of the following problems? Use "√" to indicate your answer**					

**Over the last 2 weeks, how often have you been bothered by any of the following problems?**	*Not at all (0)*	*Several days (1)*	*More than half the days (2)*	*Nearly every Day (3)*
1. **Little interest or pleasure in doing things**				
2. **Feeling down, depressed, or hopeless**				
3. **Trouble falling or staying asleep, or sleeping too much**				
4. **Feeling tired or having little energy**				
5. **Poor appetite or overeating**				
6. **Feeling bad about yourself or that you are a failure or have let yourself or your family down**				
7. **Trouble concentrating on things, such as reading or watching television**				
8. **Moving or speaking so slowly that other people could have noticed.**				
9. **Thoughts that you would be better off dead, or of hurting yourself**				
**Total**				
**Social Isolation**				
6-Item Loneliness Scale	Yes (2)		More or less (1)	No (0)
1. There are plenty of people I can rely on when I have problems a				
2. There are many people I can trust completely a				
3. There are enough people I feel close to a				
4. I miss having people around				
5. I experience a general sense of emptiness				
6. I often feel rejected				

If. Valuation of Lost Productivity Questionnaire (VOLP) – BASELINE*: http://www.thevolp.com/*

**Unpaid work**	
**The following questions ask about unpaid work in the past 7 days, not including today. If you did not perform a particular activity, please simply write '0' hours.**	
**1.** During the past 7 days, how many hours have you spent on:	**Number of hours in the past 7 days**
Housework (e.g. preparing meals, cleaning the house, washing clothes)	_______ hours
Shopping (e.g. shopping for the daily groceries, other types of shopping, going to the bank or post office)	_______ hours
Odd jobs and chores (e.g. house repairs, gardening, fixing the car)	_______ hours
Doing things for or with your own children (e.g. caring for them, taking them to school, helping with homework)	_______ hours
Voluntary activities	_______ hours
**Total time spent on these unpaid work activities**	_______ hours
**2.** During the past 7 days, have you had help with any of your household tasks (cleaning the house, shopping, taking care of the children) due to YOUR HEALTH? (Please think of any physical, mental, or emotional problems or symptoms; **tick all that apply.**)	**Number of hours in the past 7 days**
1. No, I have performed my household tasks myself	
2. Family members (e.g. partner, children) have taken over my household tasks	_______ hours
3. Others (e.g. neighbours or volunteers) have taken over my household tasks	_______ hours
4. I have had a home-help	_______ hours
5. I have had another type of paid help	_______ hours

**Employment status**	
**3.** Which of the following best describes your current employment status (tick one only)?<br>1. Working full time as an employee **(SKIP TO QUESTION 6)**<br>2. Working part time as an employee **(SKIP TO QUESTION 6)**<br>3. Self-employed **(SKIP TO QUESTION 6)**<br>4. On official work disability<br>5. Unemployed but looking for work<br>6. Unemployed but not looking for work<br>7. Retired<br>8. Housewife / househusband<br>9. Other (please specify) ____	
**4.** Is your current unemployment status mainly due to YOUR HEALTH? (Please think of any physical, mental, or emotional problems or symptoms.) A. YES B. NO	
**5.** Do you feel well enough to work if a job is available?<br>1. YES, I am able to work full time **(END OF QUESTIONNAIRE)**<br>2. YES, but I am only able to work part time **(END OF QUESTIONNAIRE)**<br>3. NO, I am unable to work at all **(END OF QUESTIONNAIRE)**	
**Job characteristics**	
If you have more than one job, please report only on your main job — the job at which you spend the majority of work hours.	
**6.** Please state your job title. (e.g. primary school teacher, chartered accountant, cashier)<br>____	
**7. In the past 3 months**, which of the following best describes your work habits *(tick one only)*?<br>1. Usually sit during the day and do not walk around very much<br>2. Stand or walk quite a lot during the day but do not often have to carry or lift things<br>3. Usually lift or carry light loads, or often have to climb stairs or hills<br>4. Do heavy work or carry very heavy loads	
**8.** On average, how many **days** do you work per week at this job?	______ days per week
**9.** On average, how many **hours** do you work per week at this job?	______ hours per week

10. What is your average annual gross income (before taxes) from paid work or self-employment? *(If you have more than one job, please report only on your main job.)*
1. Less than 12,000
2. 12,000 – 18,000
3. 18,000 – 23,000
3. 23,000-30,000
4. 30,000 or more
0. I do not know or I prefer not to answer

11. What kind of business, industry or service is your working organization? (e.g. construction, primary school, hospital, police, farm, shoe shop, food wholesale, factory)

____

**Absenteeism (absence from work)**	
**12.** In the past 3 months, how many work days **in total** have you been absent from work because of YOUR HEALTH (any physical, mental, or emotional problems or symptoms)? *Please include* ***work days*** *you missed due to your health, and/or* ***partial work days*** *where you went in late or left early due to your health (e.g. doctor appointments).* ***Do not*** *include any work days you missed to participate in this study.*	_______ work days
**Work performance**	

13. In the past 7 days, have you gone to work? 1. YES 2. NO **(IF NO, SKIP TO QUESTION 17)**

14. Think of all the work you have completed **during the past 7 days**. Would you complete the same work in **less time** if you did NOT experience any health problems *(i.e., any physical, mental, or emotional problems or symptoms)?* 1. YES 2. NO **(IF NO, SKIP TO QUESTION 16)**

15. If yes, please indicate the time you took to complete all your work in the past 7 days and the time you would take to complete the same work if you did NOT experience any health problems:

a) Time taken to complete all of my work during the past 7 days	______ hours
b) Time I would take to complete the same work if I did NOT experience any health problems (***should be less than a)***)	______ hours

16. In the past 7 days, to what extent was your performance at work affected by YOUR HEALTH **while you were working?** *(Please think of any physical, mental, or emotional problems or symptoms.)* ***Please indicate the effect on the line by marking with a cross 'X'*** **Working environment**

**My health had no effect on my work**	**0**	**1**	**2**	**3**	**4**	**5**	**6**	**7**	**8**	**9**	**10**	**I could not do any work at all due to my health**

17. During your **most recent period** of absence due to YOUR HEALTH, was your work *(tick one only):*
1. Taken over by others
2. Partly taken over by others and partly postponed until I returned
3. Postponed until I returned
0. Do not know

18. Who mainly took over your work during your **most recent period** of absence due to YOUR HEALTH *(tick one only)?*
1. Co-workers
2. Supervisors
3. Temporary worker(s)/additional staff hired from outside agencies to do my work
4. No one
0. Do not know

19. Imagine if you are at work but YOUR HEALTH affects your ability to complete your work, will your work be *(tick one only):*
1. Taken over by others
2. Partly taken over by others and partly postponed until later (i.e., I will do it later)
3. Postponed until later (i.e., I will do it later)
0. Do not know

20. If you are at work but YOUR HEALTH affects your ability to complete your work, who mainly takes over the work you cannot complete *(tick one only)?*
1. Co-workers
2. Supervisors
3. Temporary worker(s)/additional staff hired from outside agencies to do my work
4. No-one
0. Do not know

21. How often do you need to work with your co-workers as a team? *(By team, we mean 'a group of people who work/act together for a common purpose (e.g. projects and tasks)'.) (Tick one only.)*
1. None of the time **(IF NONE OF THE TIME, SKIP TO QUESTION 24)**
2. A little of the time
3. Some of the time
4. Most of the time
5. All the time

22. For the time you are working with a team, how many co-workers do you usually work with as a team? *(If you are working with more than one team,* ***please focus on the team you spend the most time with****. Please DO NOT include yourself.) Please write down a specific number such as '4' or a range such as '8–12'*
____

23. For the time you are working with a team, how important are you to the function of your team? *(If you are working with more than one team,* ***please focus on the team you spend the most time with.****) (Tick one only.)*
1. My team can **function as usual** when I am absent, or when I am present but less productive (*e.g. this might be appropriate for a person who works in a team picking crops in a field. Each person in the team picks crops all by himself or herself*)
2. My team's function can **be affected a little bit** when I am absent, or when I am present but less productive
3. My team's function can **be somewhat affected** when I am absent, or when I am present but less productive
4. My team's function can **be affected quite a lot** when I am absent, or when I am present but less productive
5. My team **cannot function** when I am absent, or when I am present for work but less productive *(e.g. this might be appropriate for the conductor of an orchestra where the orchestra can't play without the conductor and the conductor is useless without the orchestra*)

24. Can any of your co-workers do your work *(tick one only)*?
1. There are co-workers who can complete my work in **the same amount of time** as me
2. My co-workers can complete my work in **a little bit more time** than me
3. My co-workers can complete my work in **somewhat more time** than me
4. My co-workers can complete my work in **a lot more time** than me
5. None of my co-workers can do my work

25. Does your working organization hire temporary (i.e., temp) workers from external agencies who do the same or similar work as you do? 1. YES 2. NO **(IF NO, END OF QUESTIONNAIRE)**

26. Can any of the temp workers hired from external agencies do your work *(tick one only)?*
1. Temp workers can complete my work in **the same amount of time** as me
2. Temp workers can complete my work in **a little bit more time** than me
3. Temp workers can complete my work in **somewhat more time** than me
4. Temp workers can complete my work in **a lot more time** than me
5. It is impossible to find any temp workers who can do my work

THANK YOU
END OF QUESTIONNAIRE

II. Health system factors questionnaire

Institutional Capacity and Response to Non-communicable Diseases (NCDs)

**1.** Is there national NCD policy/ strategy or guideline copy in your facility? A. Yes B. No C. I don't Know		
2. Does it address the following NCD and risk factor activities/functions? You can encircle more than one.		
A) Primary prevention B) Health promotion C) Early detection/screening D) Health care and treatment E) Surveillance, monitoring and evaluation F) Capacity-building G) Palliative Care		
3. Are NCDs included in your annual facility plan? A. Yes B. No C. I don't Know		
4. Is there Health management information system available for recording patient information that includes NCD status? Yes B. No C. I don't Know		
5. If yes Q10 Is it an electronic medical records/health records system? Yes B. No C. I don't Know		
6. Indicate the availability* of the following basic technologies for early detection, diagnosis/monitoring of NCDs in the primary care facilities of the public and private health sector where: Generally available = 1; Generally, not available = 2; Don't know = 3.		
*** Generally available: in 50% or more of health-care facilities Generally not available: in less than 50% of health-care facilities**	Availability at PHC facilities in Public sector	Availability at PHC in Private sector
**Overweight and obesity**		
a) Measuring of weight		
b) Measuring of height		
**Diabetes mellitus**		
c) Blood glucose measurement		
d) Oral glucose tolerance test		
e) HbA1c test		
f) Dilated fundus examination		
g) Foot vibration perception by tuning fork		
h) Foot vascular status by Doppler		
i) Urine strips for glucose and ketone measurement		
**Cardiovascular disease**		
j) Blood pressure measurement		
k) Total cholesterol (lipid Profile) measurement		
l) Urine strips for albumin assay		
m)Serum creatinine assay		
n) Urine microalbuminuria test strips		

7. Describe the availability* of the medicines below in the primary care facilities of the public health sector, where: Generally available (above 80%) = 1; Generally, not available (below 80%) = 2; Don't know = 3.	
**Generic drug name**	Availability
**Insulin**	
**Aspirin (100 mg)**	
**Metformin**	
**Thiazide Diuretics**	
**ACE Inhibitors (Enalapril or Capitopril)**	
**CCBs (Amlodipine)**	
**Beta Blockers (Atenolol, Metoprolol etc.)**	
**Statins (Simvastatin or any)**	
**Oral morphine**	
**Sulphonyl urea(s) (Glibenclamide)**	
**Furosemide**	
**Benzathine penicillin Injection**	
**Nicotine replacement therapy**	
**Glucose 40%**	

8. Indicate the availability* of the following procedures for treating NCDs in the publicly funded health system, where: Generally available (above 50%) = 1; Generally, not available (< 50%) = 2; Don’t know = 3.	
**Procedure Name**	**Availability**
**a) Retinal photocoagulation**	
**b) Renal replacement therapy by dialysis**	
**c) Renal replacement by transplantation**	
**d) Coronary bypass or stenting**	
**e) Thrombolytic therapy (streptokinase) for acute MI**	
9. Indicate the availability (Generally available: reaches 50% or more patients in need) of palliative care for patients with NCD in the public primary health system: A. Generally available B. Generally, not available C. Don’t know	
10. What proportion of primary health care facilities are offering cardiovascular risk stratification for the management of patients at high risk for heart attack and stroke? A. None B. < 10% C. 10-50% D. > 50% E. Don’t know	
11. If Other than none to above question: Which CVD risk scoring chart is used? A. WHO/ISH risk prediction charts B. Others (specify) _________________ C. Don’t know	
12. What percentage of public sector health facilities have provision for care of acute stroke and rehabilitation? A. None B. Less than 10% C. 10% to 50% D. More than 50% E. Don’t know	
13. Rehabilitation for stroke patients 1. Generally available 2. Generally not available 3. I don’t Know	
14. Is there stroke awareness creation campaign? A. Yes B. No C. I don’t Know	
15. Is there a register of patients who have had rheumatic fever and rheumatic heart disease? A. Yes B. No C. I don’t Know	
16. If yes to Q15: Are there systems for follow-up to deliver long-term penicillin prophylaxis? A. Yes B. No C. I don’t Know	
17. Are there Health Education sessions held in the facility to create community awareness about NCDs risk factors? A. Yes B. No C. I don’t Know	
18. If yes to Q17, what are areas addressed in Health education programs in your facility? A. Physical inactivity B. Smoking (active and Passive) C. Harmful use of alcohol D. Saturated fat intake E. Salt Consumption F. Overweight/Obesity	
19. The following questions are related to your Institutional management system. Please encircle the actual practice.	
A. Is there Leadership for quality at all levels with in facility?	A. Yes B. No C. I am not sure
B. Is the system open and transparent for providers and service users?	A. Yes B. No C. I am not sure
C. Is there teamwork in system?	A. Yes B. No C. I am not sure
D. Is there accountability at all levels?	A. Yes B. No C. I am not sure
E. Is there a system for continuous learning in the system?	A. Yes B. No C. I am not sure
F. Is there active feedback loops for improvement?	A. Yes B. No C. I am not sure
G. Is there meaningful staff, user and community engagement?	A. Yes B. No C. I am not sure
H. Is there a system for empowering individuals?	A. Yes B. No C. I am not sure
I. Is there alignment of professional and organizational values?	A. Yes B. No C. I am not sure
J. Is there encouraging system for self-importance in care?	A. Yes B. No C. I am not sure
K. Valuing compassionate care	A. Yes B. No C. I am not sure
20. Is there hospital improvement committee?	A. Yes B. No C. I am not sure
21. If yes to Q20, who are the members of committee? (list members and their role):	
22. If yes to Q20, how frequently the committee meet? (minute evidence) A. Available B. Not available	

III. Health professional knowledge survey tool

Hello Dear Doctor

The following are questions designed to assess the comprehensive hypertension management in adults. This is part of ***Designing cost-efficient service delivery model for prevention and control of hypertension research*** and your willingness and provision of genuine information is important for service delivery model design and finally to improve hypertension care in the country. It will take 15-20 minutes of your time to complete.

S.No	**A. Sociodemographic factors and CRC Questionnaire**	
1	Name of health Facility	__________
2	Age in ___________Years	Sex: A. Male C. Female
3	Sex	________________________________
4	Years practice (experience)	
5	Qualification	
6	Number of patients visited per day	
**Professionals Knowledge on comprehensive hypertension care**		
4	Risk Factors for hypertension in Adults include; You can encircle more than one	A. Obesity B. Physical Inactivity C. Diet D. Smoking E. Alcohol F. Obstructive sleep Apnea (**OSA**) G. Thyroid disease H. Drugs
5	Target LDL cholesterol goal is	**A**. <100 mg/dL **B**. <130 mg/dL **C**. <70 mg/dL
6	Obesity is commonly managed by	**A**. Reduced calorie diets **B**. Physical activity C. Drugs
7	What is the most common type of hypertension?	A. Essential hypertension B. Secondary hypertension
8	What is/are major cases essential hypertension	A. Diabetes B. Kidney problem C. Unknown
9	What are Causes of secondary hypertension?	A. Medications B. Sleep apnea C. Thyroid disease D. Genetics
10	Screening general population for hypertension is recommended at	A. Age ≥ 18 years B. Age > 30 years C. Age > 45 years
11	Which one of these drugs can case secondary hypertension?	A. NSAIDs B. OCPs C. Steroids D. Anti-depressants E. MAO inhibitors F. Cyclosporine G. Erythropoietin H. All
12	Hypertension diagnosed by	A. Carefully taken single blood pressure reading B. Mean BP taken over several visits C. Home based BP measurement
13	Select BP measurement errors that cannot falsely increase BP:	*A.* Bladder distension *B.* Cuff too small C. Insufficient rest period *D.* Talking during measurement E. Cuff too large F. White-coat effect G. Caffeine intake before measuring H. Alcohol intake before measuring
14	Hypertension, dyslipidemia and diabetes have common risk factors	A. True B. False
15	Patients with hypertension should be screened for diabetes.	A. True B. False
16	Diabetes is diagnosed by (you can encircle more than one)	**A**. FPG ≥126 mg/dL **B.** Symptoms of hyperglycemia and RPG ≥200 mg/dL **C.** A1C level ≥6.5%
17	Glycemic goals of DM in non-pregnant adults is	**A.** A1C ≤6.5% **B**. FPG <110 mg/dL **C.** FPG <140 mg/dL and RBG <180 mg/dL in non-ICU patients
18	What is target BP for patients without diabetes or end organ damage for Age < 60 years	**A.** < 140/90 mm Hg **B.** < 130/90 mm Hg **C**. < 150/90 mm Hg
19	What is target BP for patients with diabetes	**A**. < 140/90 mm Hg **B.** < 130/90 mm Hg **C.** < 150/90 mm Hg
20	Which one is not first line drugs for treatments of hypertension without comorbidity indication?	**A.** Thiazide diuretic **B.** ACEIs **C.** Beta-blockers **D.** Long-acting DHP CCBs **E.** ARBs
21	Monotherapy is generally effective in control BP	A. True B. False

22	Non-drug therapy for hypertension **should not include:**	A. Physical activity B. Salt control C. Smoking cessation D. Reducing Alcohol Use E. Consuming fruits and vegetables F. Using saturated/trans-fats
23	'ACTIVE' is an approach to reduce physical inactivity. Can you describe it?	A. Yes B. No **If Yes** please describe it: ____________________
24	'SHAKE' is an approach for salt control. Can you describe it?	A. Yes B. No **If Yes** please describe it: ____________________
25	'REPLACE' is an approach for avoiding trans-fats. Can you describe it?	A. Yes B. No **If Yes** please describe it: ____________________
26	'MPOWER' is an approach for controlling Tobacco use. Can you describe it?	A. Yes B. No **If Yes** please describe it: ____________________
27	'ACT FAST' is an approach for stroke awareness. Can you describe it?	A. Yes B. No **If Yes** please describe it: ____________________
28	What is Opportunistic Screening for hypertension? A. Screening Adult population yearly B. Screening every individual attending health facility for hypertension C. Screening adults above 40 years D. Screening Individuals with 10-year CVD risk greater than 20%	
29	In this question you are required to select and encircle appropriate agent during treating hypertension with given concomitant disease. You can encircle more than response from given alternatives	

**Coexisting Condition Disease**	**Specific Agent**
I. Diabetes	**A. ACEI/ARB** B. Diuretics C. CCBs D. β blocker
II. Heart failure (HFrEF)	**A. ACEI/ARB B. β-Blocker** C. Non-dihydropyridine CCBs
III. Ischemic Heart disease (Angina, MI)	**A. ACEI/ARB B. β-Blocker** C. CCBs
IV. History of Stroke or TIA	**A. ACEI/ARB** B. Diuretics C. Combination of ACEIs + Thiazides
V. Chronic Renal failure	**A. ACEI/ARB** B. Diuretics C. CCBs D. β blocker
VI. Impotence	**A. ACEI/ARBs** B. CCB C. α-Blockers D. β blocker E. Thiazides
VII. Pregnancy	A. **Methyldopa** B. **CCBS** C. **labetalol** D. **Hydralazine** E. ACEI, ARB

30	In black adults with hypertension but without HF or CKD, including those with DM, initial antihypertensive treatment should include a thiazide-type diuretic or CCBs**.** **A. True** B. False	
31	Drugs that can be used for treatment hypertension emergency__. You can encircle more than one?	A. Nicardipine B. Sodium Nitroprusside C. Hydralazine D. Labetolol E. Enalaprilat (active form of Enalapril) **F**. All
32	The following are matching questions contains antihypertensive drugs in Column **'A'** and major side effects in column **'B'**. Match them in Space provided	
	**A**	**B**
I	____Diuretics A	A. Impotence, decreased libido, dizziness, lethargy, constipation, nausea, dry eye
II	____Beta-adrenoceptor antagonists B	B. Cold extremities, dizziness, fatigue, insomnia, nausea, anorexia, vivid dreams, depression, reduced verbal memory
III	___Central alpha-2-adrenoreceptor agonists (Methyl-dopa, clonidine) C	C. Impotence, tiredness, diarrhea, dry mouth, depression, vivid dreams, sleep disturbance, postural hypotension, sedation, reduced verbal memory
IV	___Alpha-1 adrenoreceptor Antagonists D	D. Postural hypotension, headache
V	___Dihydropyridine CCBs E	E. Ankle edema, flushing, headache, dizziness
VI	____Non-dihydropyridines F	F. Constipation, headache, nausea, dizziness
VII	____ACE inhibitors G	G. Cough, rash, taste disturbance, angioedema
33	The following questions are about hypertension and related comorbidities, and relative indications and contra-indications for anti-hypertensive medications: Please encircle from given alternatives.	
I	Thiazides are relatively contra-indicated in patients with hypertension and____: **A**. Gout B. Urinary incontinence C. Heart failure D. CKD	
II	Non-dhydroperidine (verapamil) CCBs are relatively contra-indicated in patients with hypertension and____: **A**. Heart failure B. Asthma C. CKD D. Osteoporesis	
III	Non-Cardioselective beta blockers are contra-indicated in patients with hypertension and____: A. Heart failure **B**. Asthma C. CKD D. Osteoporesis	

IV. Tool for assessing Clinical performance and quality measures for adults with high blood pressure

**Clinical performance and quality measures**	***Name of Hospital :*** ____________
Performance measures for HBP	
1. Percentage of Patients 18 to 85 years of age who had a diagnosis of Stage 2 HTN and whose SBP was <140 mm Hg during the year	
2. Percentage of Patients 18 to 85 years of age who had a diagnosis of a Stage 2 HTN and whose SBP Was <130 mm Hg during the year	
3. Percentage of patients 18 to 85 years of age who had a diagnosis of stage 1 HTN and whose SBP was <130 mm Hg during the year	
4. Percentage of patients 18 to 85 years of age who had a diagnosis of either stage 1 or 2 HTN and whose SBP Was <130 mm Hg during the year	
5. Percentage of adults 18 to 85 years of age who had a diagnosis of Stage 2 HTN who have documentation of a discussion of Intensive Lifestyle Modification (*ILM*) with their healthcare providers during the Year	
6. Use of HBPM for management of ACC/AHA Stage 2 HBP	
Process Quality measures	
7. Percentage of adults 18 to 85 years of age who had a diagnosis of elevated BP who have a documented discussion of ILM in ≥1 visits during the year	
8. Percentage of adults 18 to 85 years of age who had a diagnosis of stage 1 HTN who have a documented discussion of ILM in ≥1 visits during the year	
9. Percentage of adults 18 to 85 years of age who had a diagnosis of stage 2 who have a documented discussion of ILM in ≥1 Visits During the Measurement Year	
10. Percentage of adults 18 to 85 years of age who had a diagnosis of Stage 1 HTN With ASCVD Risk ≥ 10% or Stage 2HTN with ≥1 prescriptions for BP medication who had ≥80% adherence to BP Medication(s) during the year	
11. Use of HBPM for management of stage 1 hypertension	
12. Use of HBPM for management of stage 1 hypertension or stage 2 hypertension	
Diagnosis, Assessment and accurate measurement	
13. Use of standard protocol to consistently and correctly measure BP in ambulatory setting	
14. Use of standard process for assessing ASCVD risk	
15. Use of standard process for properly screening all adults ≥18 years and for high BP	
16. Use of an EHR to accurately diagnose and assess HTN control	
**Patient centered approach for controlling BP**	
17. Use of standard process to engage patients in shared decision-making, tailored to their personal benefits, goals and values for evidence based interventions to improve BP control	
18. Demonstration of infrastructure and personnel that assesses and addresses social determinants of health of patients with high BP	
Implementation of a system of care for patients with high BP	
19. Use of team-based care to better manage HTN	
20. Use of telehealth, m-health, e-health and other digital technologies to better diagnose and manage HTN	
Diagnose and manage HBP	
21. Use of a single, standardized plan of care for all patients with HTN	
Performance measures to improve care for patients with HBP	
22. Use of performance and quality measures to improve quality of care for patients with HBP	

V. Patient data abstraction

	Patient data abstraction (abstract the following information's from the patient medical record)	The most recent	2nd recent	3rd recent
**1**	**Anthropometric measurements**			
	What is the documented BMI in kg/m^2			
	What is the documented Waist circumference in cm?			
**2**	**BP related**			
	What is documented BP in mmHg? (Recent 3 measurements)			
	What is the initial treatment drug given			
	What is the initial dose drug 1			
	What is the initial dose drug 2			
	What is the initial dose drug 3			
	What is the initial dose drug 4			
	When was it started?			
	Is there any treatment intensification done? If yes describe			
	Is there any drug change?			
3	**Blood glucose related**			
	What is documented FBG in mg/dL?			
	What is the documented HbA1C%?			
	Is the patient diabetic?			
	If yes what is documented type of diabetes			
	If Type 2 diabetes, what is the initial regimen started?			
	Was metformin dose intensification done?			
	What is the current dose of Metformin being taken?			
4	**Cholesterol related**			
	What is the documented LDL in mg/dL?			
	What is the documented total Cholesterol in mg/dL?			
	What is triglyceride level?			
	What is the HDL cholesterol?			
5	**Other investigations**			
	What is the documented in SCr?			
	What is the recorded BUN?			
6	**Drugs for comorbidities and dosages**			
	Comorbidity1:			
	Comorbidity 2:			
	Comorbidity 3:			
	Comorbidity 3:			
**7**	**Non-pharmacologic therapy**		**Yes**	**No**
	Documented evidence on physical exercise counseling			
	Documented evidence on Weight control/reduction			
	Documented evidence on smoking cessation counseling			
	Documented evidence on harmful alcohol use reduction counseling			
	Documented evidence on DASH counseling			
	Documented evidence on social networking to address social determinants			
8	**Health care costs**			
	Inpatient stays if there is history of hospitalization			

	Outpatient clinic visit	
	Medical Services	
	Drug acquisition,	
	Dispensing	
	Administration	
	Monitoring	
	Laboratory test	
	Imaging study costs	
	Preventive Education costs	
