# Supplementary material for: Cost-effectiveness of hypertension therapy based on 2020 International Society of Hypertension guidelines in Ethiopia from a societal perspective
Source: PLoS One. 2022 Aug 29;17(8):e0273439. doi: 10.1371/journal.pone.0273439 (PMC9423649; doi:10.1371/journal.pone.0273439)
Supplement: S2 File — (DOCX) [file pone.0273439.s018.docx]

**References**

1. Antikainen R, Jousilahti P, Tuomilehto J. Systolic blood pressure, isolated systolic hypertension and risk of coronary heart disease, strokes, cardiovascular disease and all-cause mortality in the middle-aged population. Journal of hypertension. 1998;16(5):577-83.
2. Ford ES, Giles WH, Mokdad AH. The distribution of 10-year risk for coronary heart disease among US adults: findings from the National Health and Nutrition Examination Survey III. Journal of the American College of Cardiology. 2004;43(10):1791-6.
3. Collaborators GRF. Global, regional, and national comparative risk assessment of 84 behavioural, environmental and occupational, and metabolic risks or clusters of risks for 195 countries and territories, 1990–2017: a systematic analysis for the Global Burden of Disease Study 2017. Lancet (London, England). 2018;392(10159):1923.
4. Flint AC, Conell C, Ren X, Banki NM, Chan SL, Rao VA, et al. Effect of systolic and diastolic blood pressure on cardiovascular outcomes. New England Journal of Medicine. 2019;381(3):243-51.
5. Rapsomaniki E, Timmis A, George J, Pujades-Rodriguez M, Shah AD, Denaxas S, et al. Blood pressure and incidence of twelve cardiovascular diseases: lifetime risks, healthy life-years lost, and age-specific associations in 1·25 million people. The Lancet. 2014;383(9932):1899-911.
6. Lloyd-Jones DM, Larson MG, Beiser A, Levy D. Lifetime risk of developing coronary heart disease. The Lancet. 1999;353(9147):89-92.
7. Global, regional, and national age-sex-specific mortality for 282 causes of death in 195 countries and territories, 1980-2017: a systematic analysis for the Global Burden of Disease Study 2017. Lancet (London, England). 2018;392(10159):1736-88.
8. Huffman MD, Mohanan PP, Devarajan R, Baldridge AS, Kondal D, Zhao L, et al. Effect of a Quality Improvement Intervention on Clinical Outcomes in Patients in India With Acute Myocardial Infarction: The ACS QUIK Randomized Clinical Trial. Jama. 2018;319(6):567-78.
9. Bundy JD, Li C, Stuchlik P, Bu X, Kelly TN, Mills KT, et al. Systolic Blood Pressure Reduction and Risk of Cardiovascular Disease and Mortality: A Systematic Review and Network Meta-analysis. JAMA Cardiology. 2017;2(7):775-81.
10. Petty GW, Brown RD, Jr., Whisnant JP, Sicks JD, O'Fallon WM, Wiebers DO. Survival and recurrence after first cerebral infarction: a population-based study in Rochester, Minnesota, 1975 through 1989. Neurology. 1998;50(1):208-16.
11. Health MSf. International Medical Products Price Guide: 2015 edition. 2015.
12. Lee SE, Lee HY, Cho HJ, Choe WS, Kim H, Choi JO, et al. Clinical Characteristics and Outcome of Acute Heart Failure in Korea: Results from the Korean Acute Heart Failure Registry (KorAHF). Korean circulation journal. 2017;47(3):341-53.
13. Choi DJ, Han S, Jeon ES, Cho MC, Kim JJ, Yoo BS, et al. Characteristics, outcomes and predictors of long-term mortality for patients hospitalized for acute heart failure: a report from the korean heart failure registry. Korean circulation journal. 2011;41(7):363-71.
14. Steg PG, Bhatt DL, Wilson PWF, D’Agostino R, Ohman EM, Röther J, et al. One-Year Cardiovascular Event Rates in Outpatients With Atherothrombosis. Jama. 2007;297(11):1197-206.
15. Lin JK, Moran AE, Bibbins-Domingo K, Falase B, Pedroza Tobias A, Mandke CN, et al. Cost-effectiveness of a fixed-dose combination pill for secondary prevention of cardiovascular disease in China, India, Mexico, Nigeria, and South Africa: a modelling study. The Lancet Global health. 2019;7(10):e1346-e58.
16. Robinson TG, Potter JF, Ford GA, Bulpitt CJ, Chernova J, Jagger C, et al. Effects of antihypertensive treatment after acute stroke in the Continue Or Stop post-Stroke Antihypertensives Collaborative Study (COSSACS): a prospective, randomised, open, blinded-endpoint trial. The Lancet Neurology. 2010;9(8):767-75.
17. Katsanos AH, Filippatou A, Manios E, Deftereos S, Parissis J, Frogoudaki A, et al. Blood Pressure Reduction and Secondary Stroke Prevention. Hypertension. 2017;69(1):171-9.
18. Yasui D, Asayama K, Ohkubo T, Kikuya M, Kanno A, Hara A, et al. Stroke Risk in Treated Hypertension Based on Home Blood Pressure: the Ohasama Study. American Journal of Hypertension. 2010;23(5):508-14.
19. Smolina K, Wright FL, Rayner M, Goldacre MJ. Long-Term Survival and Recurrence After Acute Myocardial Infarction in England, 2004 to 2010. Circulation: Cardiovascular Quality and Outcomes. 2012;5(4):532-40.
20. Butler J, Kalogeropoulos AP, Georgiopoulou VV, Bibbins-Domingo K, Najjar SS, Sutton-Tyrrell KC, et al. Systolic blood pressure and incident heart failure in the elderly. The Cardiovascular Health Study and the Health, Ageing and Body Composition Study. Heart. 2011;97(16):1304.
21. Piller LB, Baraniuk S, Simpson LM, Cushman WC, Massie BM, Einhorn PT, et al. Long-term follow-up of participants with heart failure in the antihypertensive and lipid-lowering treatment to prevent heart attack trial (ALLHAT). Circulation. 2011;124(17):1811-8.
22. Davis BR, Kostis JB, Simpson LM, Black HR, Cushman WC, Einhorn PT, et al. Heart Failure With Preserved and Reduced Left Ventricular Ejection Fraction in the Antihypertensive and Lipid-Lowering Treatment to Prevent Heart Attack Trial. Circulation. 2008;118(22):2259-67.
23. Moita B, Marques AP, Camacho AM, Leão Neves P, Santana R. One-year rehospitalisations for congestive heart failure in Portuguese NHS hospitals: a multilevel approach on patterns of use and contributing factors. BMJ open. 2019;9(9):e031346.
24. Chamberlain AM, Dunlay SM, Gerber Y, Manemann SM, Jiang R, Weston SA, et al. Burden and Timing of Hospitalizations in Heart Failure: A Community Study. Mayo Clinic proceedings. 2017;92(2):184-92.
25. Law MR, Morris JK, Wald NJ. Use of blood pressure lowering drugs in the prevention of cardiovascular disease: meta-analysis of 147 randomised trials in the context of expectations from prospective epidemiological studies. BMJ (Clinical research ed). 2009;338:b1665.
26. Asayama K. Observational study and participant-level meta-analysis on antihypertensive drug treatment-related cardiovascular risk. Hypertension Research. 2017;40(10):856-60.
27. Cherry SB, Benner JS, Hussein MA, Tang SSK, Nichol MB. The Clinical and Economic Burden of Nonadherence with Antihypertensive and Lipid-Lowering Therapy in Hypertensive Patients. Value in Health. 2009;12(4):489-97.
28. Liu L, Wang Z, Gong L, Zhang Y, Thijs L, Staessen JA, et al. Blood pressure reduction for the secondary prevention of stroke: a Chinese trial and a systematic review of the literature. Hypertension Research. 2009;32(11):1032-40.
29. Stenberg K, Lauer JA, Gkountouras G, Fitzpatrick C, Stanciole A. Econometric estimation of WHO-CHOICE country-specific costs for inpatient and outpatient health service delivery. Cost Effectiveness and Resource Allocation. 2018;16(1):11.
30. Organization WH. WHO-CHOICE Estimates of Cost for Inpatient and Outpatient Health Service Delivery.
31. Salomon JA, Vos T, Hogan DR, Gagnon M, Naghavi M, Mokdad A, et al. Common values in assessing health outcomes from disease and injury: disability weights measurement study for the Global Burden of Disease Study 2010. Lancet (London, England). 2012;380(9859):2129-43.
32. Organization WH. Disability weights, discounting and age weighting of DALYs. Available; 2016.
33. Salomon JA, Haagsma JA, Davis A, de Noordhout CM, Polinder S, Havelaar AH, et al. Disability weights for the Global Burden of Disease 2013 study. The Lancet Global Health. 2015;3(11):e712-e23.
34. Vos T, Allen C, Arora M, Barber RM, Bhutta ZA, Brown A, et al. Global, regional, and national incidence, prevalence, and years lived with disability for 310 diseases and injuries, 1990–2015: a systematic analysis for the Global Burden of Disease Study 2015. The Lancet. 2016;388(10053):1545-602.
35. ICF C. Ethiopia Demographic and Health Survey 2016, Addis Ababa, Ethiopia, and Rockville, Maryland, USA: CSA and ICF. DF-1.6.
36. Institute. EPH. Ethiopia steps report on risk factors for chronic non-communicable diseases and prevalence of selected NCDs. 2016.
37. DESA U. World Population Prospects 2019: Highlights. New York (US): United Nations Department for Economic and Social Affairs. 2019.
38. Kelemu Tilahun Kibret, Mesfin YM. Prevalence of hypertension in Ethiopia: a systematic meta-analysis. . Public Health Reviews 2015;36(14).
39. WHO. Non-communicable diseases country profiles 2018. Geneva: World Health Organization. 2018.
40. Kuriakose A, Nair Anish TS, Soman B, Varghese RT, Sreelal TP, Mendez AM, et al. Rate and Risk of All Cause Mortality among People with Known Hypertension in a Rural Community of Southern Kerala, India: The Results from the Prolife Cohort. Int J Prev Med. 2014;5(5):596-603.
41. Dicker D, Nguyen G, Abate D, Abate KH, Abay SM, Abbafati C, et al. Global, regional, and national age-sex-specific mortality and life expectancy, 1950–2017: a systematic analysis for the Global Burden of Disease Study 2017. The lancet. 2018;392(10159):1684-735.
42. Ko MJ, Jo AJ, Park CM, Kim HJ, Kim YJ, Park D-W. Level of blood pressure control and cardiovascular events: SPRINT criteria versus the 2014 hypertension recommendations. Journal of the American College of Cardiology. 2016;67(24):2821-31.
43. Sorato MM, Davari M, Kebriaeezadeh A, Sarrafzadegan N, Shibru T, Fatemi B. Risk of fatal and nonfatal coronary heart disease and stroke events among adult patients with hypertension: basic Markov model inputs for evaluating cost-effectiveness of hypertension treatment: systematic review of cohort studies. Journal of Pharmaceutical Health Services Research. 2021;12(2).
44. Gu Q, Dillon CF, Burt VL, Gillum RF. Association of Hypertension Treatment and Control With All-Cause and Cardiovascular Disease Mortality Among US Adults With Hypertension. American Journal of Hypertension. 2010;23(1):38-45.
45. Murakami Y, Hozawa A, Okamura T, Ueshima H. Relation of Blood Pressure and All-Cause Mortality in 180 000 Japanese Participants. Hypertension. 2008;51(6):1483-91.
46. Nagai K, Yamagata K, Iseki K, Moriyama T, Tsuruya K, Fujimoto S, et al. Antihypertensive treatment and risk of cardiovascular mortality in patients with chronic kidney disease diagnosed based on the presence of proteinuria and renal function: A large longitudinal study in Japan. PLoS One. 2019;14(12):e0225812.
47. Gudmundsson LS, Johannsson M, Thorgeirsson G, Sigfusson N, Sigvaldason H, Witteman JCM. Risk profiles and prognosis of treated and untreated hypertensive men and women in a population-based longitudinal study The Reykjavik Study. Journal of Human Hypertension. 2004;18(9):615-22.
48. Law M, Wald N, Morris J. Lowering blood pressure to prevent myocardial infarction and stroke: a new preventive strategy. Health technology assessment (Winchester, England). 2003;7(31):1-94.
49. Law MR, Wald NJ, Morris JK, Jordan RE. Value of low dose combination treatment with blood pressure lowering drugs: analysis of 354 randomised trials. BMJ (Clinical research ed). 2003;326(7404):1427.
